# The RNA N^6^-methyladenosine methylome coordinates long non-coding RNAs to mediate cancer drug resistance by activating PI3K signaling

**DOI:** 10.1038/s41419-025-08045-6

**Published:** 2025-11-07

**Authors:** Yanhong Tan, Changli Zhou, Sicheng Bian, Huiqin Bian, Yanan Ren, Wencke Walter, Jiuxia Pang, Tao Cheng, Hongwei Wang, Yuchao Yang, Wenzheng Guo, Lingli Zhang, Aref Al-Kali, Mark R. Litzow, Xiaonan Han, Jianhua Yu, Rendong Yang, Gang Huang, Gregor Hoermann, William Tse, Shujun Liu

**Affiliations:** 1 Department of Medicine, The MetroHealth System, Case Western Reserve University, Cleveland, OH USA; 2 Department of Hematology, The Second Hospital of Shanxi Medical University, Taiyuan, Shanxi China; 3 Robert H. Lurie Medical Research Center, Chicago, IL USA; 4 MLL Munich Leukemia Laboratory, Munich, Germany; 5 Division of Hematology, Mayo Clinic, Rochester, MN USA; 6 Division of Hematology & Oncology, Department of Medicine, School of Medicine, Chao Family Comprehensive Cancer Center, University of California, Orange, California USA; 7 Department of Cell Systems and Anatomy, UT Health San Antonio, Joe R. and Teresa Lozano Long School of Medicine, San Antonio, Texas USA; 8 Gene and Cell Therapy Institute (GCTI), The MetroHealth System, Case Western Reserve University, 2500 MetroHealth Dr, Cleveland, OH USA

**Keywords:** Molecular biology, Haematological cancer

## Abstract

Long non-coding RNAs (lncRNAs) and RNA N⁶-methyladenosine (m^6^A) have been linked to leukemia drug resistance. However, whether and how lncRNAs and m^6^A coordinately regulate resistance remain elusive. Here, we show that many differentially expressed lncRNAs enrich m^6^A, and more lncRNAs tend to have higher m^6^A content in CML cells resistant to tyrosine kinase inhibitors (TKIs). We demonstrate the broad clinical relevance of our findings, showing that upregulation of top-ranked lncRNAs (e.g., SENCR, PROX1-AS1, LINC00892) in TKI-resistant cell lines occurs in CML patients at the diagnostic stage, blast crisis phase, or not responding to TKIs compared to the chronic phase or TKI responders, respectively. Higher lncRNAs predict drug resistance and shorter survival duration. The knockdown of SENCR, PROX1-AS1, or LINC00892 restores TKI sensitivity. Mechanistically, upregulation of PROX1-AS1, SENCR, and LINC00892 results from FTO-dependent m^6^A hypomethylation that stabilizes lncRNA transcripts and empowers resistant cell growth through overexpression of PI3K signaling mediators (e.g., ITGA2, F2R, COL6A1). Treatment with PI3K inhibitor alpelisib eradicates resistant cells in vitro and in vivo, with prolonged survival of leukemic mice through downregulation of F2R, ITGA2, and COL6A1. Thus, the lncRNA-m^6^A-PI3K cascade represents a new non-genetic predictor for drug resistance and poorer prognosis in cancer, and a pan-cancer mechanism underlying TKI resistance.

## Introduction

The constitutively activated tyrosine kinases (TKs) are hallmark features of several types of cancer, such as chronic myeloid leukemia (CML) and Ph+ ALL. It is well known that the growth and progression of CML is attributed to a hybrid protein BCR::ABL1 [1], resulting from the t(9;22)(q34;q11) chromosomal translocation. Multiple TK inhibitors (TKIs), such as imatinib, nilotinib, dasatinib, bosutinib, and ponatinib, have emerged as leading compounds to treat CML. Initial clinical responses of CML patients to TKIs are frequently positive [2–5]. However, relapse and disease progression characterized by drug resistance occurs even with continuous drug administration, and patients with TKI-resistant CML may still succumb to their disease [6–12]. Studies regarding TKI resistance in leukemia have mostly centered on cancer genetic alterations [13, 14], for example, acquired mutations (which happen in about 1/3 of progressive cases) in the kinase domains of BCR::ABL1 that reduce or even fully impair TKIs’ binding to ABL kinase. However, non-genetic factors are emerging as essential regulators for TKI resistance, which remain largely undefined.

*N*^*6*^-methyladenosine (m^6^A) is the most common internal modification on RNAs, including long non-coding RNAs (lncRNAs). The m^6^A is installed by a methyltransferase complex [15, 16], erased by the demethylases (e.g., FTO) [17–20], and recognized by m^6^A-binding proteins [21]. Cumulative evidence suggests that m^6^A methylation occurs frequently in a dynamic and reversible manner [22–27], and significantly regulates RNA half-life, RNA splicing, and protein translation [28–31], thus determining target expression levels. Abnormal m^6^A methylation disrupts various biological processes, implicated in the initiation, development, and progression of cancers [32–36]. However, it is less known whether and how dynamic and reversible RNA epitranscriptomic mechanisms play a role in TKI resistance, particularly for resistant patients without acquired mutations. In the current work, we tested the hypothesis that epitranscriptomic diversity in heterogeneous tumor cell populations may generate divergence in the expression of cell fate determination genes/pathways that can swiftly avoid drug-induced cell death. We recently demonstrated that a dynamic FTO/m^6^A axis emerges as a key epigenetic driver of reversible TKI-tolerance state, making a contribution to acquired TKI resistance through activating cell proliferation/anti-apoptotic genes [37]. Yet, it is not well understood whether and how the FTO/m^6^A axis empowers leukemia cells to survive prolonged drug exposure through non-protein-coding genes.

LncRNAs are transcripts with lengths of ≥200 nucleotides and have no apparent protein-coding potential. As they crucially activate or repress transcription of protein-coding genes [38], lncRNA dysregulations have been linked to cancer progression and drug resistance [39–47]. Previous studies suggest that functions of lncRNAs are subjected to m^6^A regulation through, for example, potentiating lncRNAs-mediated transcriptional repression [48, 49]. These findings, together with the observations that upregulation of certain lncRNAs fuels the survival and proliferation of cancer cells [50–52], raise the possibility that, upon exposure to TKIs, the dynamic m^6^A methylation allows a set of proliferation/anti-apoptosis-relevant lncRNAs bearing m^6^A motifs to be rapidly upregulated, thus helping a subpopulation of cells avoid TKI killing. In the present work, we examined the potential interactions between the m^6^A methylome and lncRNAs in resistant leukemia cells and found that this interaction shapes the balance between TKI resistance and sensitivity. We dissected its pathological relevance in leukemia patients and found that m^6^A-upregulated lncRNAs are novel biomarkers associated with TKI non-responders and predict worse outcomes.

## Materials and methods

### Mice

All animal experiments were approved by the Institutional Animal Care and Use Committees of the University of Minnesota and were in accordance with the US National Institutes of Health Guide for Care and Use of Laboratory Animals. For survival studies, mice were sacrificed when they showed any signs of distress (i.e., breathing disorders, weight loss, or immobility).

The NOD/SCID/γcnull immunodeficient NOG mice (female, male; 4–6 weeks old) were purchased from Charles River and sublethally irradiated. About 0.5 × 10^6^ nilotinib-resistant K562 cells were injected into the irradiated mice through the tail-vein 4 h after irradiation. Recipient mice were monitored weekly for signs of leukemia beginning on day 7 after transplantation. Mononuclear cells collected from bone marrow (BM) of the euthanized mice were stained with mouse anti-human CD45 antibody (BioLegend #368509) for flow cytometry sorting. The presence of a CD45+ population was considered as human leukemia engraftment. The development of leukemic disease was verified by H&E staining. Then, BM cells were isolated for further investigations.

For preclinical testing of PI3K inhibitor: Alpelisib was prepared just prior to administration by dissolving it in DMSO to provide a clear solution and then diluting with 2-Hydroxypropyl-β-cyclodextrin (HPCD) and PBS (ratio 2.5:75:22.5). About 0.5 × 10^6^ BM cells isolated from the leukemic mice will be injected into the sublethally irradiated NSGS mice (second recipient). The leukemia-bearing mice were randomized and administered alpelisib intraperitoneally every 3 days at 100 mg/kg for 5 weeks. The administration of vehicle (DMSO, HPCD, PBS; ratio 2.5:75:22.5) was used as a negative control. The survival time was analyzed by Kaplan–Meier estimate, and comparison between groups was analyzed by log-rank tests. The survival time was from the start of the leukemia cell injection. Cytospin preparations of BM cells were processed for Wright-Giemsa staining. The lungs, spleens, and livers were immediately fixed in 10% neutral-buffered formalin and stained with H&E.

### AML and CML patient samples

The current study was approved by the Institutional Review Board of Mayo Clinic and the second hospital of Shanxi Medical University and conducted in accordance with the Declaration of Helsinki. The diagnoses of acute myeloid leukemia (AML) and CML were made according to the criteria of the World Health Organization. Mononuclear cells from BM or PB samples of AML and CML patients were prepared by Ficoll-Hypaque (GE Healthcare #71-7167-00) gradient centrifugation. The patient cells were frozen in 10% DMSO plus 90% FBS and directly used for molecular biological assays without further cell culture. All patients signed an informed consent document approved by the Institutional Review Board before entering any study.

### Cell lines and cell culture

Leukemia cell lines, K562, MV4-11, and Kasumi-1, were newly purchased from American Type Culture Collection with no further authentication or testing for mycoplasma. Cell lines were grown in RPMI-1640 (GE Healthcare #SH30027.01) supplemented with 20% (Kasumi-1) or 10% (K562, MV4-11) fetal bovine serum (FBS, Gibco by Life Technologies^TM^ #16140-071) and Antibiotic-Antimycotic (Gibco by Life Technologies^TM^ #15240062) at 37 °C under 5% CO_2_. No cell line used in this paper is listed in the database of commonly misidentified cell lines maintained by ICLAC (International Cell Line Authentication Committee).

### Plasmid design and construction

Three shRNAs against *FTO* (TRCN0000183897, TRCN0000179651, TRCN0000180978) and the negative control vectors (pLKO.1) were obtained from BMGC RNAi (University of Minnesota), but the shRNA against lncRNAs PROX1-AS1 (NR_037850.2; AS7, AS1521, AS3299), SENCR (NR_038908.1; S16, S785, S1238) and LN892 (NR_038461.1; LN370, LN619, LN576) were designed using the online tool (https://portals.broadinstitute.org/gpp/public/seq/search), synthesis by Integrated DNA Technologies Inc, and cloned into pLKO.1 vector. The primers are listed in Supplementary Table 7.

### In vitro adaptation of TKI-resistant cells

Cell lines, K562, Kasumi-1, and MV4-11, were passaged with low concentration of imatinib or nilotinib (0.1 µM) and sequentially cultured in increasing concentrations of these TKIs (0.3, 1 µM) for 8–10 weeks. Cells cultured in parallel in drug-free medium were used as parental/sensitive controls. Cells were considered resistant when they could routinely grow in medium containing 1 µM imatinib or nilotinib, respectively.

### Lentivirus vector, virus production, and virus infection

For virus production, HEK-293 (3.8 × 10^6^) cells were planted in a 10 cm cell culture dish for 24 h, and transfected with 6 µg of target or scrambled control plasmids using calcium phosphate transfection reagent (CalPhos™ Mammalian Transfection Kit), following the manufacturer’s instructions. The lentiviruses were harvested at 48 and 72 h after transfection and concentrated using the protocol of the Lenti-X™ Concentrator (Clotech #631232). For virus infection, leukemia cells (1 × 10^6^) were infected by the lentiviruses using Polybrene (final concentration 4 µg/ml) in 1 ml medium, and puromycin (final concentration 2 µg/ml) was added to select the stable transformants 24 h post-infection.

### Colony-forming assays

Colony-forming assays were performed using MethoCult® medium (Stem Cell Technologies #03434) as previously reported [37, 53, 54]. Briefly, the cells were suspended in 0.3 ml of IMDM medium (Stem Cell Technologies #36150), mixed with 3 ml MethoCult® medium, and then dispensed into 35 mm dishes. The colony counts and sizes were recorded after 7–10 days. Generally, 10 single clones were picked up into 96-well plates for each cell line to get the stable clone after virus infection and puromycin selection.

### m^6^A dotblotting

The mRNA was extracted using GenElute™ direct mRNA Miniprep Kit (Sigma #DMN70-1KT). The m^6^A RNA dotblotting was performed with a Bio-Dot Apparatus (Bio-Rad #170-6545). About 500 ng mRNA was diluted in 50 µl RNase-free water and mixed with 150 µl RNA incubation solution (1 ml mix: 657 µl formamide, 210 µl 37% formaldehyde solution, and 133 µl 10× MOPS). Then the RNA was denatured at 65 °C for 10 min and mixed with 200 µl ice-cold 20× SSC. The RNA loading membrane was baked at 80 °C for 5 min, UV crosslinked, blocked with 5% non-fat milk at room temperature for 45 min, and incubated with m^6^A antibody overnight. After being washed 3 times with 1× PBST, the membranes were incubated with an HRP-conjugated secondary antibody anti-rabbit in 5% non-fat dry milk. The signal was detected by enhanced chemiluminescence. RNA spotted membrane was stained with 0.02% methylene blue (Sigma #1808) in 0.5 M sodium acetate (pH 5.0) for loading control. The antibodies used are listed in Supplementary Table 6.

### Western blotting

The whole cellular lysates were prepared by harvesting the cells in 1× cell lysis buffer [20 mM HEPES (pH 7.0), 150 mM NaCl, 0.1% NP40] supplemented with 1 mM phenylmethane sulfonyl fluoride (PMSF, Sigma #10837091001), 1× Phosphatase Inhibitor Cocktail 2 and 3 (Sigma #P5726, P0044), and 1× protease inhibitors (protease inhibitor cocktail set III, Calbiochem-Novabiochem #539134). The proteins were resolved by sodium dodecyl sulfate (SDS)–polyacrylamide gel electrophoresis, transferred onto PVDF membranes (GE Healthcare #10600023), blocked by 5% non-fat milk, followed by probing with first and HRP-conjugated secondary antibodies (listed in Supplementary Table 6).

### RNA isolation, cDNA preparation, and quantitative PCR (qPCR)

According to the manufacturer’s instructions, the total RNA was isolated using miRNeasy Kit (Qiaqen #217004), and complementary DNA (cDNA) synthesis was performed using SuperScript® III First-Strand Synthesis System (Invitrogen #18080-051). The expression of target genes was assessed by SYBR Green qPCR (Applied Biosystems #4309155). The expression of the targets was analyzed using the ΔCT approach. The levels of *GAPDH or 18s* were used as normalization in cell lines, but ABL was used as an internal control in patient samples according to *BCR::ABL1* IS. The primers are listed in Supplementary Table 7.

### m^6^A immunoprecipitation (IP)

The RNAs were diluted in 200 µl IPP buffer (150 mM NaCl, 0.1% NP40, 10 mM Tris-HCl, pH 7.4) and fragmented into 100-nucleotide-long fragments using sonication. The fragmented RNAs were incubated for 12 h at 4 °C with 5 µl anti-m^6^A (listed in Supplementary Table 6) in IPP buffer. The mixture was then immunoprecipitated by incubation with Dynabeads™ Protein G (ThermoFisher #10004D) at 4 °C for an additional 3 h. After extensive washing by IPP buffer, 75 µl 42 °C pre-heated Elution Buffer (0.02 M DTT, 0.15 M NaCl, 0.05 M Tris-HCl, pH 7.4, 0.001 M EDTA, 0.1% SDS) was added into the m^6^A-positive RNA solution for 5 min at 42 °C, and this step was repeated 2 times. Finally, the enriched m^6^A RNA was eluted from the beads into 225 µl solution and precipitated by adding 2.5 times the volume of 100% ethanol.

### Cell proliferation and apoptosis assays

Cell proliferation assays were performed using Cell Counting Kit-8 (CCK-8, Dojindo Molecular Technologies #CK04-11) following the manufacturer’s instructions. Briefly, the parental and resistant cells with various treatments (1.5 × 10^4^) in RPMI-1640 medium (100 µl) were dispensed into 96-well flat-bottomed microplates and incubated for 24 h. The cells were cultured for another 24 or 48 h, and the CCK-8 reagent (10 µl) was added to each well. The microplates were incubated at 37 °C for an additional 2–4 h. Absorbance was read at 450 nm using a microplate reader, and the results were expressed as a ratio of the treated over untreated cells (as 100%). Five wells were sampled per experimental group in each experiment. Cell apoptosis assays were performed using Annexin V-PI Apoptosis Detection Kit I (BD Pharmingen^TM^ #556547) according to the manufacturer’s instructions and followed by flow cytometry analysis.

### Identification of differentially expressed annotated lncRNAs

For all IP and IN samples, the RNA-seq raw reads were aligned to the UCSC human reference genome (GRCh38/hg38) by Hisat2 [55] with parameter –U and then sorted and indexed by SAMtools [56]. The mapped reads were counted using featureCounts v1.6.1 [57], and a differential expression analysis comparing resistance and parental samples was performed using R packages, edgeR [58] and limma [59]. The differentially expressed lncRNAs (annotated by GENCODE v19) with an absolute value of log_2_FC > 0 and FDR < 0.05 were considered for future analysis. The top 40 most differentially expressed lncRNAs having m^6^A sites in nilotinib samples were selected from the upregulated lncRNAs, and a heatmap of these lncRNAs ranked by log_2_FC in decreasing order was plotted by the R package pheatmap.

### Peak reads count analysis for m^6^A sequencing

Peaks were called for each of the three groups by using the MeTPeak R package as described previously and were further filtered by choosing only those assigned to annotated lncRNAs. For lncRNAs that have more than one peak, the mean of the fold enrichment of all their peaks was used as their fold enrichment. To compare m^6^A enrichment of lncRNAs between resistant and parental samples, the lncRNAs occurred only in one sample were assigned 0-fold enrichment in the other sample, and then log_2_ (resistant fold enrichment)—log_2_ (parental fold enrichment) was calculated to obtain log_2_ Fold Change (if one of these two-fold enrichment values is 0, add 1 to both before log_2_ transformation), finally, R package ggplot_2_ was used to plot a histogram for the log_2_ Fold Change calculated above.

### Hematoxylin and eosin (H&E) staining

Mouse tissues (lung, liver, spleen) were fixed in 10% neutral-buffered formalin, deparaffinized, hydrated, and stained with H&E (Thermo-Scientific), which was performed at the image center, the MetroHealth System, Case Western Reserve University.

### Cytospin/Wright-Giemsa staining

The mouse BM cells (0.1 × 10^6^) were isolated and placed in the Shandon EZ Single Cytofunnel (Thermo Electron Corporation). Samples were centrifuged at 1000 rpm for 8 min. The slides were air-dried and stained with Hema-3 Kit (22-122-911, Fisher Scientific, Hampton, NH). Stained slides were viewed and photographed using a Leica microscope mounted with a high-resolution spot camera with Image-Pro Plus software. Morphologic differentiation was determined by calculating the percentage of post-mitotic cells containing metamyelocytes, bands, and segmented neutrophils within six visual fields per slide (magnification ×200).

### Analysis of Gene Expression Omnibus (GEO) data and functional pathways

LncRNA and gene expression profile data were downloaded and analyzed for the expression of lncRNAs. These samples were normalized, managed, and analyzed by GraphPad Prism 5 Software. Further, pathway and gene-enrichment analyses that are associated with SENCR expression were conducted using DAVID 6.8 (KEGG_pathway) software. The significance of the association between target genes and canonical pathway was computed using two parameters: (1) a ratio of the number of target genes from the dataset that map to the pathway divided by the total number of genes that constitute the canonical pathway; and (2) a −log_10_ (*P* value) determining the probability that the association between the DEGs in the dataset and the canonical pathway is due to chance alone. In the present analysis, the computed −log_10_ (*P* value) of 3.0 (*P* < 0.05) and above was considered statistically significant.

### Analysis of the MLL database

The cohort comprised a total of 985 patients: 64 healthy individuals, 774 AML patients, and 121 CML patients at initial diagnosis. Furthermore, sequential samples of 12 CML patients with progression from chronic to blast phase CML were analyzed. BM or peripheral blood (PB) samples from these patients had been sent to the MLL Leukemia Laboratory between 2006 and 2023 for diagnostic work-up. The respective diagnosis was established based on cytomorphology, immunophenotyping, cytogenetics, and molecular genetics following WHO guidelines. AML patients were grouped according to the European Leukemia Net (ELN) 2022 risk group [60]. All patients gave their written informed consent for scientific evaluations. The study was approved by the Internal Review Board and adhered to the tenets of the Declaration of Helsinki. RNA was extracted using the MagNA Pure 96 Cellular RNA LV Kit (Roche LifeScience, Mannheim, Germany). For transcriptome analysis, the TruSeq Total Stranded RNA kit was used, starting with 250 ng of total RNA, to generate RNA libraries following the manufacturer’s recommendations (Illumina). 2 × 100 bp paired-end reads were sequenced on the NovaSeq 6000 (Illumina) with a median of 50 million reads per sample. Using BaseSpace’s RNA-seq Alignment app (v2.0.1) with default parameters, reads were mapped with the STAR aligner (v2.5.0a) to the human reference genome hg19 (RefSeq annotation). Estimated read counts for each gene were normalized by applying the trimmed mean of M-values normalization method, and the resulting log_2_ counts per million were used.

### Statistical analysis

In statistical analysis, we used *n* ≥ 3, and checked the normality vs non-normally distributed data. We use the Mann–Whitney U tests and non-parametric tests when it is non-normally distributed data. All analyses were performed using the GraphPad Prism 5 Software. *P* < 0.05 was considered statistically significant. All *P* values were two-tailed. No blinding or randomization was used. No samples or animals were excluded from analysis. All criteria were pre-established. No statistical method was used to predetermine sample size, and the sample size for all experiments was not chosen with consideration of adequate power to detect a pre-specified effect size. Variations were compatible between groups. In vitro experiments, such as qPCR, Western blotting, cell proliferation assays, dotblotting, etc., were routinely repeated three times unless indicated otherwise in figure legends or main text. For every figure, the statistical tests were justified as appropriate.

## Results

### The activities of the targeted kinase signaling are not required for the survival and proliferation of TKI-resistant cells

**Fig. 1:**
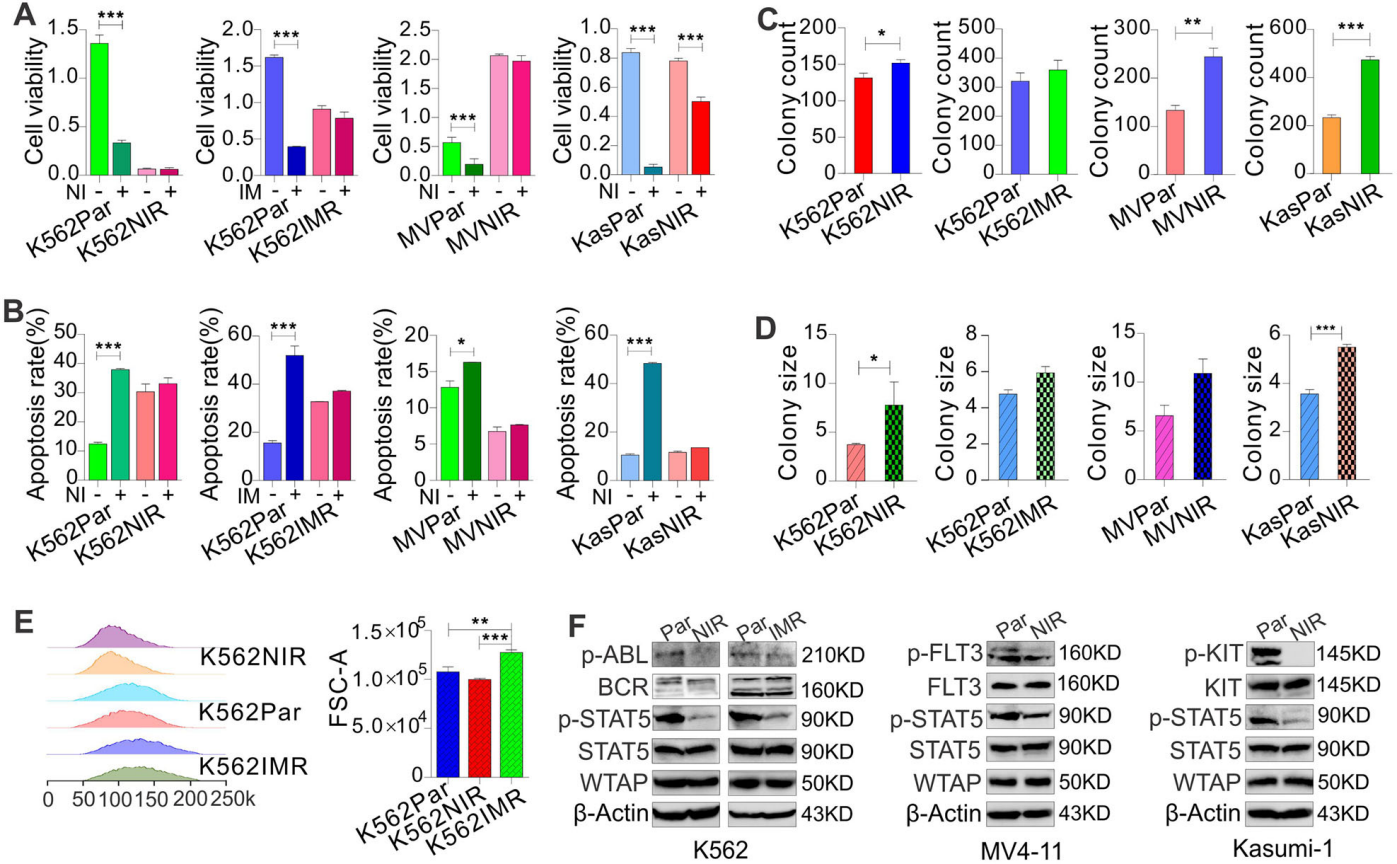
Generation and characterization of leukemia cells with acquired resistance to nilotinib or imatinib. **A**, **B** CCK-8 assays for cell proliferation and flow cytometry assays for cell apoptosis in parental and resistant cells treated with 1 µM nilotinib or imatinib for 72 h. The data of CCK-8 represent two independent experiments with 6 repeats in total, but the flow cytometry assays represent three independent experiments. **C**, **D** Colony-forming assays for K562, Kasumi-1, and MV4-11 parental and resistant cells in drug-free medium. **E** Flow cytometry assays to measure cell size in K562 resistant and parental cells. **F** Western blotting of parental and resistant cells growing in drug-containing medium. In (**A**–**E**), data are expressed as mean ± S.E.M. of triplicate samples. In (**F**), the data are representative of three independent experiments. Par parental, NIR nilotinib resistant, IMR imatinib resistant, NI nilotinib, IM imatinib, MV MV4-11, Kas Kasumi-1. *P < 0.05, **P < 0.01, ***P < 0.001.

To dissect the molecular mechanisms of acquired resistance to inhibitors of the TK pathway, we generated drug-resistant derivatives of leukemia cells K562 (BCR::ABL1), MV4-11 (FLT3), and Kasumi-1 (c-KIT) by long-term culture in the presence of TKIs nilotinib (second generation) or imatinib (first generation) (10, 30, 100, 300, 1,000 nM). Cells cultured in parallel without drugs serve as parental/sensitive controls. Cells are considered resistant when they can routinely grow in medium containing 1 µM nilotinib or imatinib, respectively. We then characterized the resistant phenotypes by measuring the survival rate of parental and resistant cells upon transient exposure to TKIs. All parental controls displayed a significant decrease in cell viability (Fig. 1A) and an increase in apoptotic cells (Figs. 1B and S1A), but the resistant cells could proliferate in the drugs with no obvious changes in cell apoptosis. Results from colony-forming assays were heterogeneous (Figs. 1C, D and S1B), in which nilotinib-resistant K562, Kasumi-1, and MV4-11 cells have significantly higher colony-forming potential with more colonies and larger colony sizes. However, imatinib-resistant K562 cells did not show significant changes in colony number and size when compared to parental cells. Flow cytometry detected different cell sizes between parental and resistant K562 cells, in which imatinib-resistant cells are the largest (Fig. 1E). Finally, we examined the activities of the targeted TKs, and found that, compared to parental cells, the phosphorylation of KIT, FLT3 and BCR::ABL1 is decreased without obvious changes in total protein expression when growing in drug-containing medium, leading to dephosphorylation of STAT5 (Fig. 1F), a shared downstream signaling mediator. These results suggest that survival and proliferation of TKI-resistant cells are independent of the targeted TK signaling.

### Transcriptome-wide m^6^A sequencing unveils distinct lncRNAs in nilotinib-resistant versus sensitive leukemia cells

**Fig. 2:**
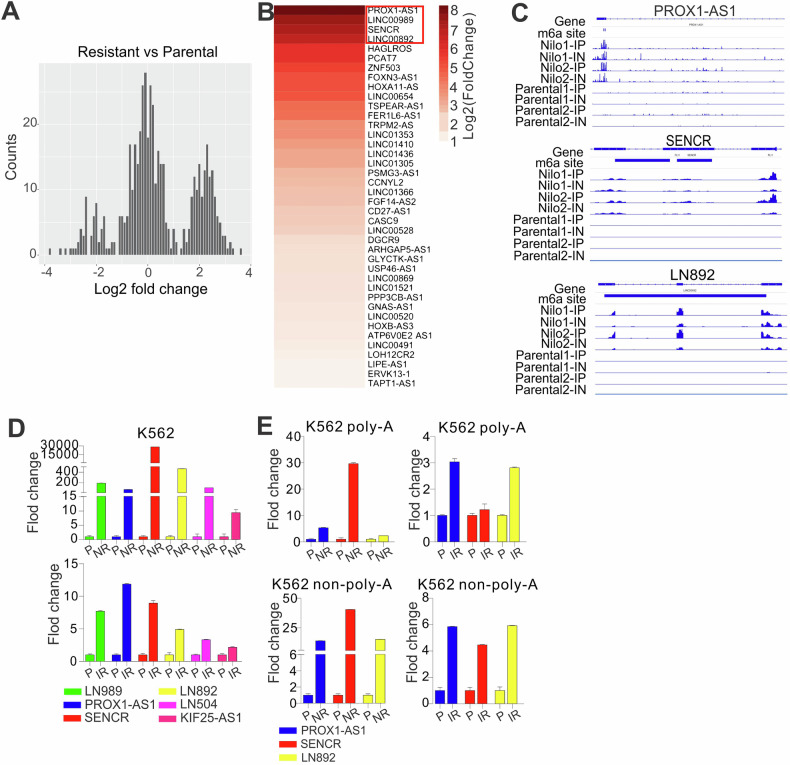
Differentially expressed lncRNAs in nilotinib-resistant cells bear m^6^A motifs. **A** Plot distribution of log_2_ change of enrichment across m^6^A peaks from parental to nilotinib-resistant cells. The average log_2_-transformed normalized signal for the duplicated m^6^A-seq was used to generate a histogram of read count values. **B** The heatmap based on the sorted log_2_ fold change of 40 upregulated lncRNAs. Forty differentially expressed lncRNAs (annotated by gencode v19) are ranked by log_2_ (fold change) with different m^6^A methylation levels. **C** LncRNAs PROX1-AS1, SENCR, and LN892 with the m^6^A-seq track are highlighted. **D** qPCR of total RNA for the expression of indicated lncRNAs in parental and resistant K562 cells. **E** qPCR for lncRNA expression in poly-A and non-poly-A RNA of K562 P, IR, and NR cells. Data is expressed as mean ± S.E.M. of duplicate samples from three independent experiments. P parental, NR nilotinib resistant, IR imatinib resistant, IP immunoprecipitation, IN input, Nilo nilotinib.

Our previous studies showed that, compared with parental cells, most changes of m^6^A peaks in resistant cells came from IncRNAs as well as CDs and 3′UTR, with less profound changes at 5′UTR [37]. These findings imply that m^6^A-associated lncRNAs may partially affect the development of TKI resistance. To test this, first, we analyzed the m^6^A-peak fold enrichment from normalized read counts mapped to the peak region, focusing on the changes of lncRNAs. We found that the distribution of log_2_ transformed fold change show three peaks, the highest peak in the middle part of the distribution indicating that most lncRNAs have comparable m^6^A levels with log_2_ fold change close to 0, while the peaks of the positive log_2_ fold change located on the right side of the distribution are much higher than those of the negative log_2_ fold change on the left side, indicating that relative more lncRNAs tend to have higher m^6^A in resistant cells (Fig. 2A). These findings imply that lncRNAs-specific m^6^A contents are increased, although the fold enrichment of overall m^6^A peaks is decreased [37], in resistant vs parental cells. Second, we analyzed m^6^A RNA sequencing data [37] and identified 315 upregulated and 97 downregulated lncRNAs (annotated; Supplementary Table 1) in resistant versus parental cells. By intersection analyses of differentially expressed lncRNAs with changed m^6^A peaks (Fig. S2A), we identified 40 upregulated lncRNAs bearing m^6^A sites in Nil (nilotinib resistance) samples (Supplementary Table 2). The heatmap in Fig. 2B was plotted based on the sorted log_2_ fold change of the top 40 overlapped lncRNAs. For all immunoprecipitation (IP) and input (IN) samples, the sorted BAM files along with m^6^A site coordinate information stored in bed files were loaded into Integrative Genomics Viewer to compare expression levels of interested intergenic lncRNAs (e.g., LINC00892 (LN892)) across different conditions (Fig. 2C).

We conducted qPCR for total RNA from K562 cells, and verified the alterations of top-ranked lncRNAs (Figs. 2D and S2B), for example, upregulation of LINC00989 (LN989), PROX1-AS1, SENCR, LN892, LN504, and KIF25-AS1; downregulation of LN659, VPS9D1-AS1, LNC200, UCA1, and WASIR2; but no change in LN1270 and MAP3K14-AS1. Given that our RNA-seq is done in the fraction of poly-A-associated RNAs, and as lncRNAs have both poly-A and non-poly-A-tailed RNAs [61], we separated poly-A-tailed RNAs from non-poly-A-tailed RNAs, and measured the lncRNA levels. We observed that the expressions of PROX1-AS1, LN892 and SENCR are upregulated as a similar pattern in both poly-A and non-poly-A-tailed fraction (Fig. 2E). Pathway analysis (Fig. S3) revealed that PROX1-AS1 regulated pathways are mostly negatively enriched, but LN892 and SENCR-regulated pathways are mostly positively enriched; pathways in red boxes are positively enriched pathways regulated by LN892 and SENCR; MYC pathway in blue box is significantly negatively enriched and regulated by all three lncRNAs; PI3K-AKT/mTOR signaling is negatively enriched and regulated by PROX1-AS1 but has trends toward being enriched when regulated by SENCR and LN892. Taken together, these data suggest that m^6^A-associated lncRNAs are involved in a non-genetic mechanism that underlies the emergence and maintenance of acquired TKI resistance.

### Expression of the m^6^A-associated lncRNAs is particularly elevated in leukemia patients at diagnosis or not responding to TKIs, and could serve as an independent predictor for poor prognosis

**Fig. 3:**
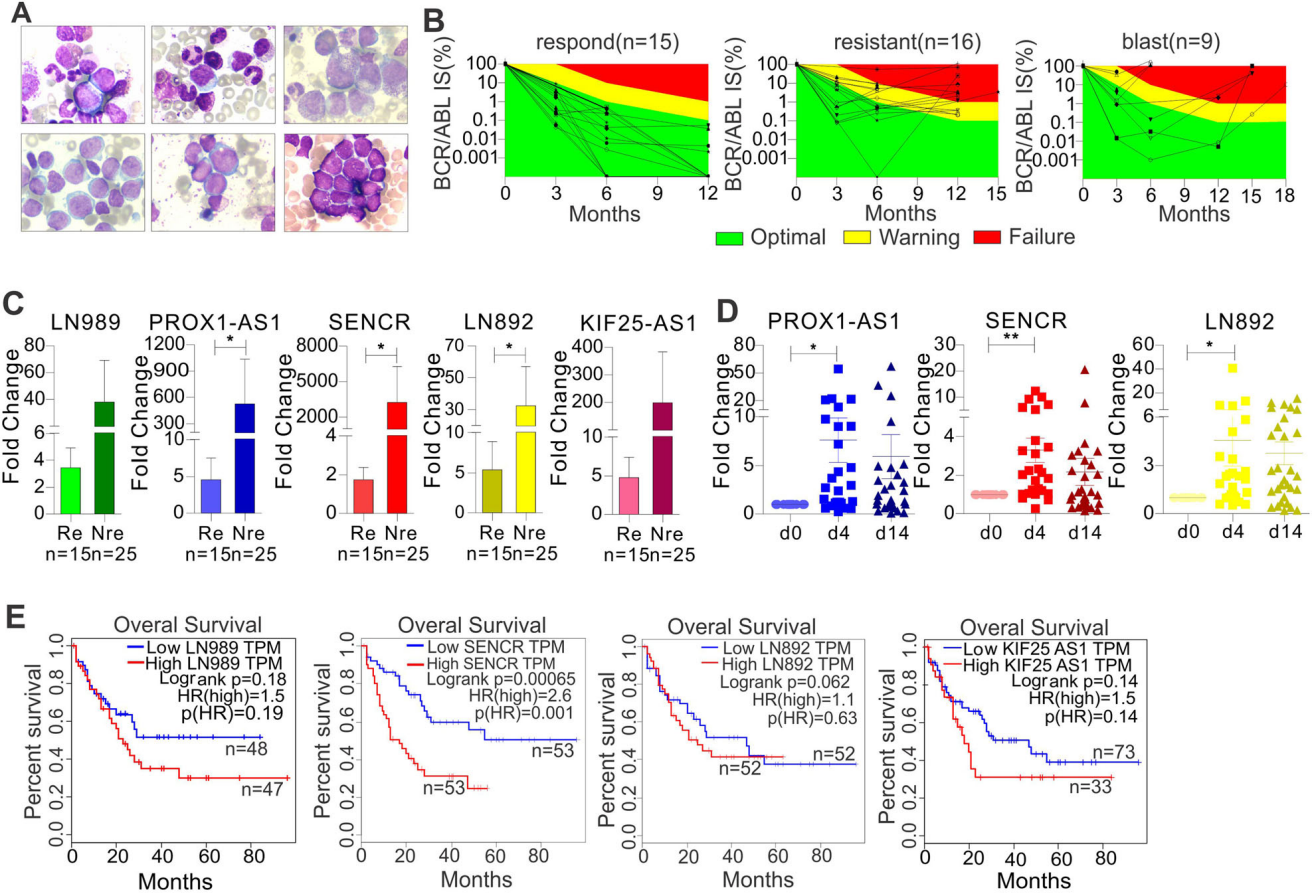
lncRNA upregulation predicts unfavorable outcomes in leukemia patients. **A** BM cell morphology of CML patients. The classical morphological images were obtained from CML-CP and CML-BP patients. Bone marrow cells were cytospinned onto slides, followed by Wright-Giemsa staining for 20 min. The images were obtained under the light microscope. Upper, chronic phase; lower, blast phase (AML and ALL). **B** Changes in the number of *BCR::ABL1* transcripts prior and post nilotinib or imatinib therapy. Green, yellow, and red areas represent optimal, warning, and failure results, respectively. **C** qPCR for lncRNA expression in CML (*n* = 40) patients who are classified as responders and inadequate responders. Median values are depicted by horizontal lines. **D** qPCR of RNA extracted from AML patients (*n* = 30) receiving nilotinib therapy. **E** The association of lncRNA expression with overall survival (OS) in leukemia patients was analyzed by the Kaplan–Meier estimate. Re responder, Nre non-responder. *P < 0.05, **P < 0.01.

To explore the clinical implications of the dysregulated lncRNAs, first, we analyzed the gene expression profiling of AML collected at diagnosis, and compared the expression of FTO, LN892, LN989, PROX1-AS1, and SENCR among different response groups. In these AML patients, a trend towards higher levels of expression of LN989 and SENCR was observed in the ELN risk groups intermediated and adverse (Fig. S4A). In line, expression of SENCR at diagnosis was higher in AML patients who did not achieve complete remission (CR) after induction chemotherapy (Fig. S4B). Furthermore, we performed an exploratory analysis of matched samples from CML patients at diagnosis in chronic phase, during hematologic remission, and at the time of progression to blast phase CML (Fig. S4C). Expression of FTO was highest at the blast phase and lowest at hematologic remission. Also, differential expressions of lncRNAs were overserved in this cohort. Such heterogeneous correlation between the expression of given genes and drug responses could be attributed to the variations of blast percentage, genetic mutations, age, and sex, which will be validated in larger cohorts of patients. To further clarify the role of FTO, LN892, LN989, PROX1-AS1, and SENCR in drug resistance, we obtained CML PB cells from patients after nilotinib or imatinib therapy. The patient characteristics are described in Supplementary Table 3. The definition of responding and inadequately responding was based on the number of *BCR::ABL1* transcripts post-therapy, but the chronic phase (Fig. 3A, upper panel; *n* = 3) versus blast crisis (Fig. 3A, lower panel; *n* = 3) was determined by cell morphology and relative clinical information. When the BCR::ABL1 international score (IS) meets response milestones at 3, 6, and 12 months (≤10% BCR::ABL1 IS at 3, ≤ 1% *BCR::ABL1* IS at 6 months and ≤0.1% *BCR::ABL1* IS at ≥12 months) post-therapy, these patients are classified as TKI responders, and others as inadequate responders (Fig. 3B) [62]. We then performed qPCR in normal and CML patient cells for LN989, PROX1-AS1, SENCR, LN892, and KIF25-AS1, which are consistently elevated in resistant K562 cells. While PROX1-AS1, SENCR, and KIF25-AS1 had a trend toward upregulation, both LN989 and LN892 had statistically significant upregulation (Fig. S4D). We also employed online tools GEPIA [63] and BloodSpot [64] to further validate the expression patterns of the aforementioned lncRNAs in leukemia patients and normal donors. Consistently, the expressions of LN892 and SENCR in GEPIA, as well as PROX1-AS1 and KIF25-AS1 in Bloodspot (Leukemia MILE Study), are much higher in leukemia patients than those in normal donors (Fig. S5A, B), with barely or undetectable expression of LN892 in both datasets. When compared with TKI responders, CML patients with inadequate response had significantly higher expressions of LN989, PROX1-AS1, SENCR, LN892, and KIF25-AS1 (Fig. 3C). When compared with chronic disease, patients with blast crisis had a trend toward higher lncRNA expression (Fig. S5C, D). Notably, to strengthen the conclusion, we employed both 18S and ABL as internal controls to normalize lncRNA expressions in patients. The same conclusions were made when using ABL, the most cited internal control, although a more obvious difference was observed when 18S was used (Fig. S5E).

To further substantiate the clinical significance of the m^6^A-associated lncRNAs in leukemia, we also examined the expression of LN989, PROX1-AS1, SENCR, LN892, and KIF25-AS1 in AML patients, who received nilotinib twice daily after induction and consolidation chemotherapy [37]. We observed that nilotinib (in combination with chemotherapy) upregulates LN989, PROX1-AS1, SENCR, LN892, and KIF25-AS1 (Fig. 3D). Finally, we used the online tool GEPIA [63] to explore the association between the expression of these lncRNAs and patient survival. As expected, overexpression of LN989, SENCR, LN892, and KIF25-AS1 significantly or has a trend to predict shorter survival time (Fig. 3E). Moreover, higher levels of LN989, SENCR, LN892, and KIF25-AS1 tend to predict poorer survival in leukemia patients from the TCGA study using two different comparisons (Fig. S6). The Kaplan–Meier test was not performed on PROX1-AS1 due to its extremely low expression in TCGA leukemia patients (median of FPKM < 0.003). Together, these results indicate that upregulation of m^6^A-associated lncRNAs [23, 24] could be a common vulnerability and prognostic factor in CML and AML patients post TKI therapy.

### LncRNA upregulation in resistant cells takes place through the enhanced RNA stability by FTO-mediated m^6^A demethylation

**Fig. 4:**
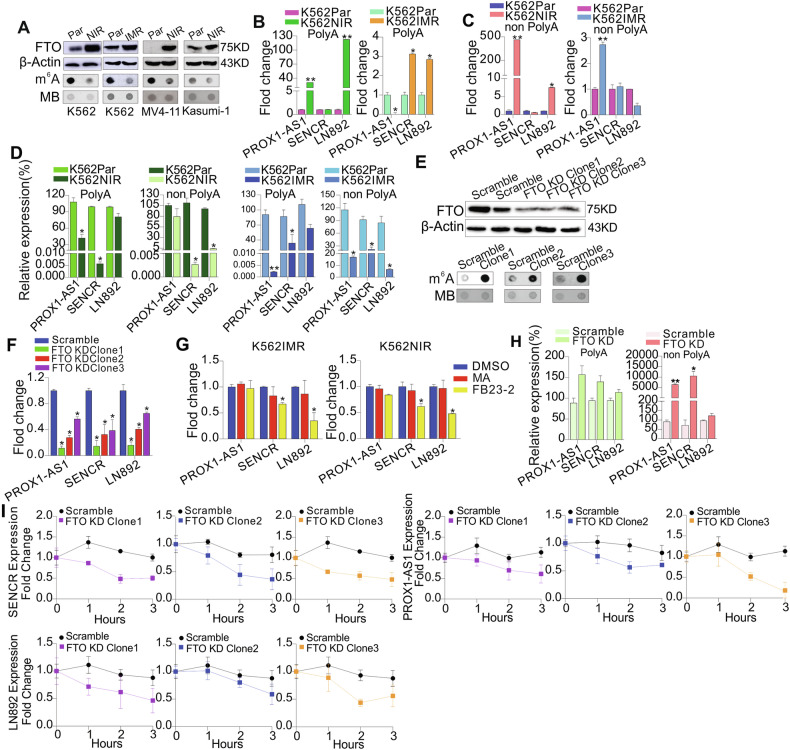
LncRNAs are partially regulated by FTO-dependent m^6^A methylation in resistant cells. **A** Dotblotting for global m^6^A amounts (lower) and Western blot for FTO protein expression (upper) in K562, MV4-11, and Kasumi-1 cells resistant to nilotinib or imatinib. The data represent three independent experiments. **B**, **C** m^6^A immunoprecipitation (IP) was performed in mRNA/poly-A (**B**) and non-poly-A RNA (**C**). The eluted RNA was converted to cDNA, and lncRNA expression was assessed by qPCR. **D** qPCR for the m^6^A-IP relative expression of lncRNAs normalized by the total levels. **E** Dotblotting for global m^6^A methylation (lower) and Western blot for FTO protein expression (upper) in clones with FTO knockdown. The data represent three independent experiments. **F** qPCR for lncRNA expression in clones with FTO knockdown. **G** qPCR for lncRNA expression in resistant cells treated with meclofenamic acid (50 µM) and FB23-2 (10 µM) for 6 h. **H** m^6^A IP was performed in mRNA and non-poly-A RNA in clones with FTO knockdown. The eluted RNA was converted to cDNA, and expression of PROX1-AS1, SENCR, and LN892, normalized by total RNA, was assessed by qPCR. **I** qPCR for lncRNA expression in scrambled and FTO knockdown clones treated with 5 μg/ml actinomycin-D for the indicated time points. The gene expression was normalized to GAPDH. In all qPCR, data are means ± S.E.M. Par parental, NIR nilotinib resistant, IMR imatinib resistant, KD knockdown.

Given that m^6^A-binding motifs are enriched within lncRNAs [37], and as m^6^A methylation has been found to regulate the stability of RNA transcripts [37, 65], lncRNA upregulation in resistant cells may result from the prolonged half-life of RNA transcripts, due to m^6^A demethylation. To this end, we first measured the levels of global m^6^A amounts and FTO expression, and observed a decrease in m^6^A abundance (Fig. 4A, lower; Fig. S7A) and an increase in FTO expression (Fig. 4A, upper) in K562, MV4-11, and Kasumi-1 cells resistant to nilotinib or imatinib, in line with our previous report [37]. Further, FTO levels were significantly elevated in nilotinib-treated AML patients and in CML patients non-responding to imatinib or nilotinib therapy (Fig. S7B). Most notably, TKI non-responders without acquired kinase domain mutations have significantly higher FTO expression than those carrying mutations. As lncRNAs include poly-A and non-poly-A tailed, we then performed RNA IP using anti-m^6^A antibody in both poly-A and non-poly-A fractions, converted the eluted RNA to cDNA, and carried out qPCR with primers covering m^6^A-binding sites. As shown in Fig. 4B, C, the m^6^A amounts for PROX1-AS1, SENCR, and LN892 were increased without obvious changes in SENCR expression in nilotinib-resistant K562 cells when using GAPDH as an internal control. For imatinib-resistant K562 cells, the amounts of PROX1-AS1 with poly-A tailed and LN892 without poly-A tailed decreased. Because the total levels of these lncRNAs are dramatically increased in resistant versus parental cells (ref. Fig. 2D, left), to accurately reflect the changes of m^6^A amount specific for these lncRNAs, we normalized the m^6^A-IP related alterations to the changes of total lncRNA levels, and observed that the m^6^A levels of PROX1-AS1, SENCR and LN892 are decreased in resistant versus parental controls, in parallel with the changes in global m^6^A content (Fig. 4D). These findings suggest that m^6^A hypomethylation accounts for lncRNA upregulation in resistant cells.

To study whether FTO, a key m^6^A demethylase [37], plays a role in lncRNA dysregulation, we infected K562 cells with scramble or FTO shRNAs (shRNA-TRCN0000183897, TRCN0000179651, TRCN0000180978) viruses, and selected TRCN0000180978 for further investigation, because TRCN0000180978 had the highest efficacy to knock down FTO (Fig. S7C). Colony-forming assays revealed that FTO knockdown impairs colony potential, as evidenced by a decrease in colony number and size (Fig. S7D). Then, single clones were selected and expanded for further investigations. Firstly, the knockdown of FTO and the increase of m^6^A methylation were confirmed in each clone (Figs. 4E and S7E). Secondly, qPCR analysis disclosed that expressions of PROX1-AS1, SENCR and LN892 are significantly inhibited in clones with FTO knockdown compared with scrambled controls (Fig. 4F). Thirdly, in line with FTO knockdown, pharmacological inhibition of FTO by meclofenamic acid (MA) [66] and FB23-2 [67] downregulated these lncRNAs in K562 cells resistant to imatinib or nilotinib (Fig. 4G). Notably, no obvious changes in FTO protein expression were observed (Fig. S7F), suggesting the enzymatic inhibition by MA and FB23-2; the more downregulation of lncRNAs by FB23-2 was consistent with its higher and more selective FTO enzymatic inhibition when compared with MA [67]. To assess changes of m^6^A on individual lncRNAs, we performed m^6^A IP in total RNAs, poly-A and non-poly-A-tailed RNAs in clones with scrambled and FTO knockdown. qPCR analysis revealed that the m^6^A content of PROX1-AS1, SENCR, and LN892 is significantly increased when normalized to the expression of lncRNAs from total RNAs (Fig. 4H). These results suggest that FTO upregulates lncRNAs by decreasing the m^6^A amount of specific lncRNAs.

It has been shown that m^6^A methylation predominantly and directly decreases transcript stability [65].To address why the m^6^A-containing lncRNAs are downregulated in FTO knockdown cells, we examined the RNA stability in FTO knockdown and control cells. Namely, we treated FTO knockdown or control clones with antinomycin-D, a transcription inhibitor [37]. The total RNA was extracted, and the lncRNA levels were assessed by qPCR, which was normalized to GAPDH. As expected, the decay of lncRNAs was shortened in FTO knockdown cells compared to scrambled cells (Fig. 4I). Collectively, these findings suggest that lncRNA upregulation in resistant cells is attributed to longer RNA half-life, which is mediated by FTO-dependent m^6^A demethylation.

### Genetic suppression of the FTO-lncRNA axis promotes nilotinib sensitivity, and decreases proliferation and growth of resistant cells

**Fig. 5:**
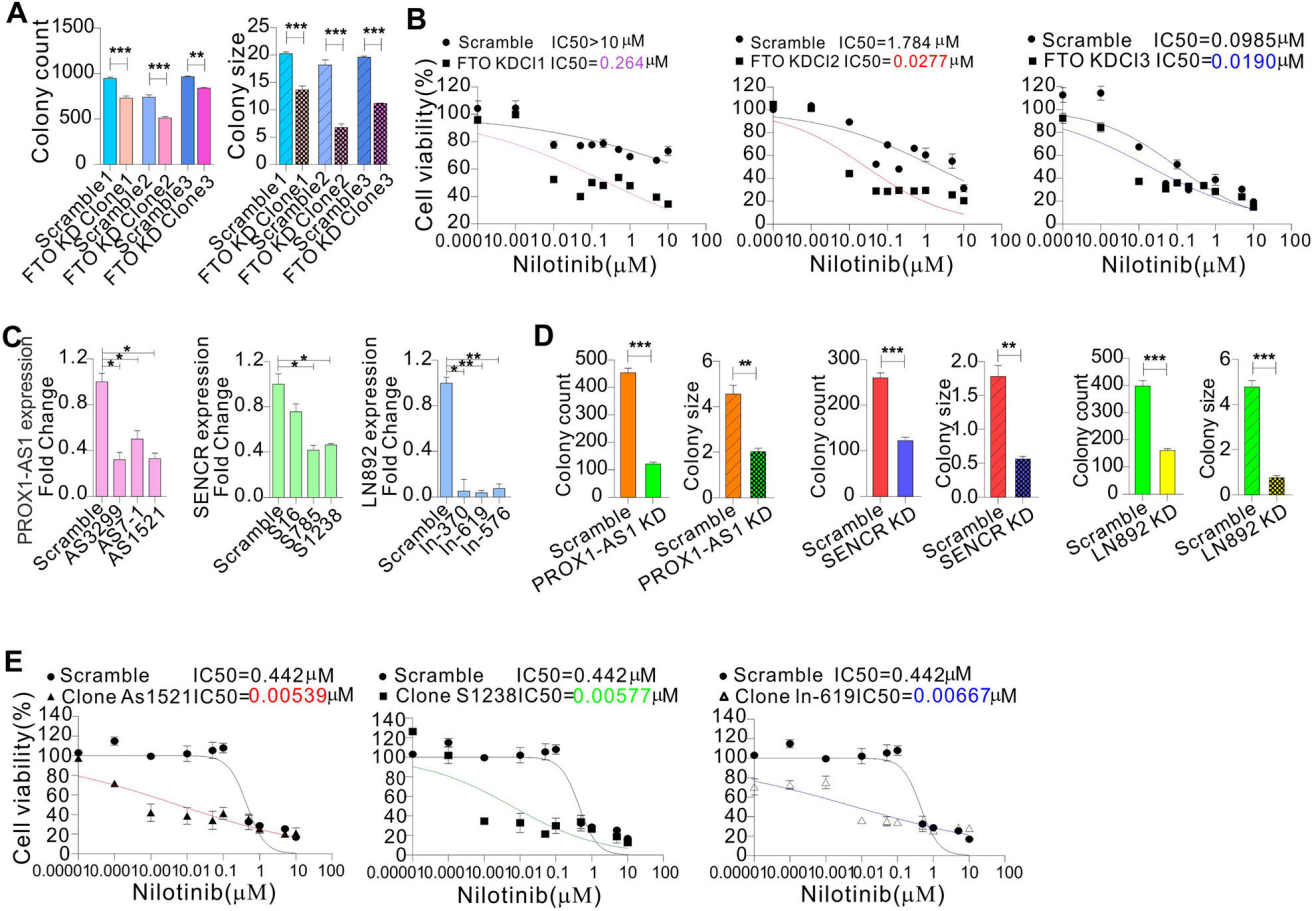
FTO-regulated lncRNAs contribute to nilotinib sensitivity. **A** Colony-forming assays in K562 clones with FTO knockdown. **B** CCK-8 assays in FTO knockdown or control clones treated with nilotinib for 48 h. The data represent two independent experiments with six repeats in total. **C** Resistant K562 cells were infected with lncRNA virus for 48 h, and qPCR was used to assess lncRNA expression. **D** Colony-forming assays in K562 resistant cells with knockdown of indicated lncRNAs. **E** CCK-8 assays in lncRNA knockdown or control clones treated with indicated doses of nilotinib for 48 h. The data represent two independent experiments with six repeats in total. KD knockdown. **P < 0.01, ***P < 0.001.

Having demonstrated the regulatory roles of FTO and m^6^A in lncRNA expression, we proceeded to address the impacts of lncRNAs on TKI sensitivity using K562 clones with stable FTO knockdown. We first confirmed that FTO knockdown impairs colony potential, as evidenced by a decrease in colony number and size compared to scrambled controls (Fig. 5A); expression of lncRNAs, including LN989, LN892, LN504, PROX1-AS1, SENCR, and KIF25-AS1, was significantly decreased in cells with FTO knockdown (Fig. S8A). We then examined the survival of FTO knockdown or scrambled clones upon transient exposure to nilotinib. As shown in Fig. 5B, the scrambled clones had IC_50_ values for nilotinib several orders of magnitude larger than those exhibited by FTO knockdown. Although all FTO knockdown clones displayed significant and dose-dependent decreases in cell viability, the scrambled clones could proliferate at drug concentrations much larger than the IC_50_ value, in agreement with our previous findings [37] that FTO positively regulates TKI sensitivity in leukemia cells.

We have shown that lncRNAs are upregulated in TKI-resistant leukemia cells and in non-responding CML patients who have worse outcomes. We hypothesized that lncRNA ablation could render resistant cells sensitive to TKIs. To test this, we knocked down LN892, PROX1-AS1, or SENCR in K562 resistant cells carrying lncRNA upregulation (Fig. 5C), because these three lncRNAs carry m^6^A motifs and are highly expressed with a marked decrease of their m^6^A methylation in resistant cells. Colony assays showed that knockdown of PROX1-AS1, SENCR, or LN892 reduces colony number and size (Figs. 5D and S8B). To test if lncRNA abundance affects nilotinib sensitivity, we treated lncRNA knockdown clones with various concentrations of nilotinib for 72 h. We found that knockdown of lncRNAs renders the resistant clone sensitive to nilotinib-inhibited cell proliferation, as supported by showing that the IC_50_ values in clones with PROX1-AS1 (AS1521), SENCR (S1238), or LN892 (ln-619) depletion are several orders of magnitude lower than those in scrambled (Fig. 5E). Collectively, these results suggest that lncRNAs enable culture leukemia cells to better withstand nilotinib-induced cell death.

### LncRNA-fueled growth of resistant cells is linked to the active PI3K-AKT signaling pathway

The function of PROX1-AS1, SENCR, or LN892 in regulating TKI resistance may be through a change in a specific gene program. To gain further insights into lncRNA-conferred resistant cell growth, we first analyzed a public database (GSE51878) using LncRNA2Target v2.0, in which SENCR was knocked down in cancer cells, to identify lncRNA downstream targets. Because the pathogenic roles of SENCR have been documented in cancer, with limited knowledge available for PROX1-AS1 and LN892, we selected SENCR, but not PROX1-AS1 and LN892, for further mechanistic study. Importantly, our findings revealed that SENCR is also subjected to regulation by FTO-dependent m^6^A hypomethylation, affects response to TKIs in vitro and in patients, and impacts patient survival. The signature of SENCR consisted of 663 differentially expressed genes (Supplementary Table 4). Using DAVID 6.8 for functional annotation, KEGG analysis provided insights into biological processes enriched in SENCR knockdown cells, and revealed many functional pathways (Supplementary Table 5), which involve 291 SENCR-regulated genes.

**Fig. 6:**
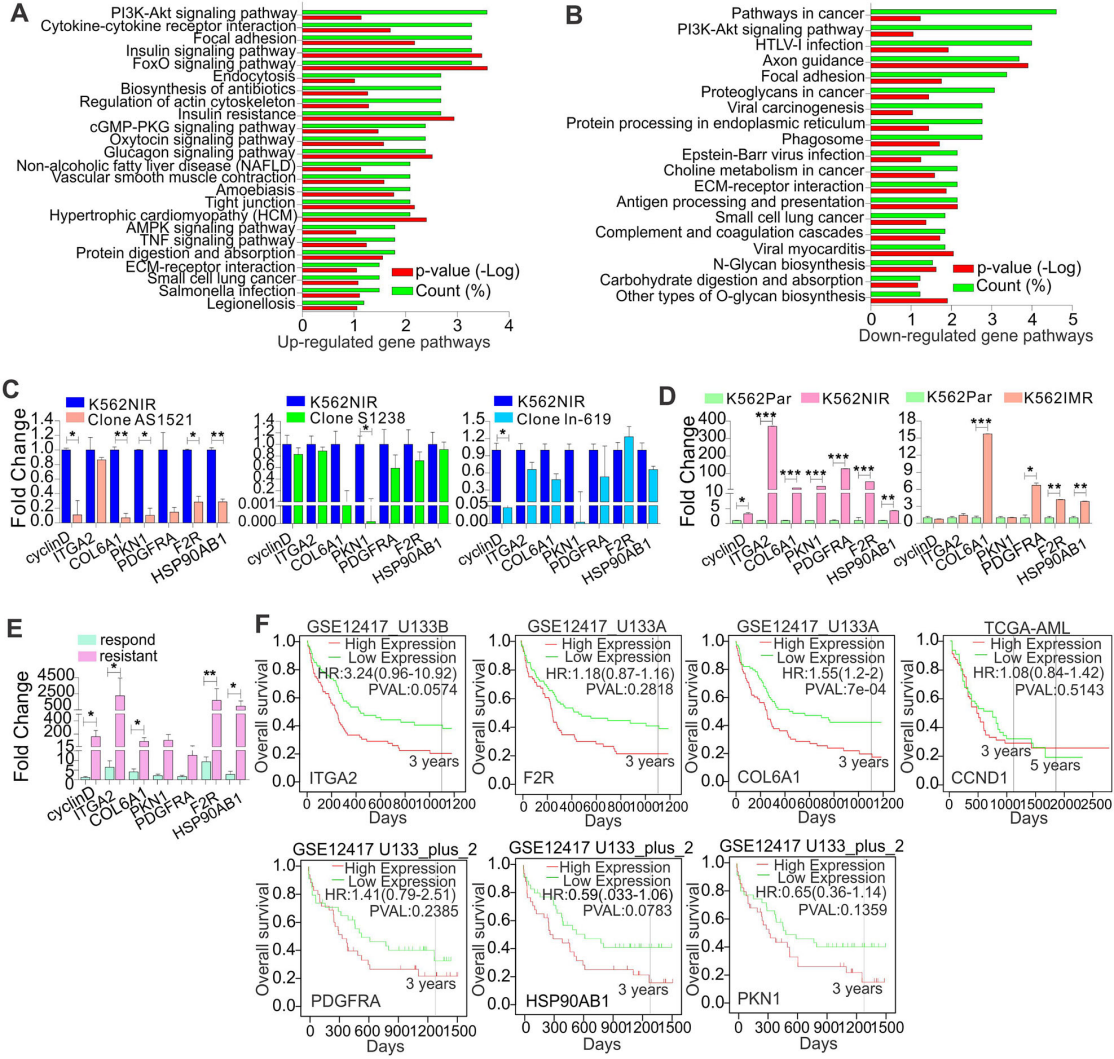
PI3K signaling pathway mediates lncRNA-sustained resistant cell growth. **A**, **B** KEGG pathway analysis identifies a network of genes related to PI3K signaling that is responsible for lncRNA-sustained cell growth. Functional pathway analysis was performed in differentially expressed genes upon SENCR knockdown using DAVID 6.8/KEGG software. Upregulated (**A**) and downregulated (**B**) gene pathways were determined by comparing SENCR knockdown with scrambled control cells. Count (%) means the ratio of genes involved in the pathway. **C** The expression of indicated genes was assessed by qPCR in K562 resistant clones with knockdown of PROX1-AS1, SENCR, or LN892. **D** The expression of indicated genes was assessed by qPCR in K562 parental and resistant (nilotinib, imatinib) cells. **E** The expressions of ITGA2, COL6A1, cyclin D1, PKN1, PDGFRA, F2R, and HSP90AB1 were assessed by qPCR in TKI responding and resistant CML patients. **F** The association of ITGA2, COL6A1, cyclin D1, PKN1, PDGFRA, F2R, and HSP90AB1 expression with overall survival (OS) in leukemia patients analyzed by the Kaplan–Meier estimate using online tool PROGgene or GEPIA2. In (**F**), the curves (ITGA2, F2R, COL6A1, cyclin D1) were adjusted for age and cohort, divided at the median of gene expression. In all qPCR, data are means ± SD.

To select the most important pathways for further investigations, we performed functional pathway analyses for all differentially expressed genes together (*n* = 663) or dividing them into up- (*n* = 337) or downregulated (*n* = 326) groups. All these strategies nominated the phosphatidylinositol 3-kinase (PI3K) pathway that mediates lncRNAs-sustained resistant cell growth (Figs. 6A, B and S9). This was especially interesting given that the PI3K signaling plays an important role in leukemia pathogenesis, with limited knowledge in drug resistance [68, 69]. Although 13 up- and 12 downregulated genes were enriched in the PI3K signaling, we focused on 7 top-ranked downregulated genes (e.g., ITGA2, COL6A1, cyclin D1, PKN1, PDGFRA, F2R, HSP90AB1), because SENCR behaved as an oncogenic lncRNA in TKI resistance. Further, these genes were either downregulated to the highest levels in cells with SENCR knockdown, or well-established resistant genes and/or annotated to have a role in sustaining cancer cell survival and proliferation (Fig. S9) [37, 70, 71]. qPCR on RNAs from independent samples confirmed that, compared with scrambled controls, ITGA2, COL6A1, cyclin D1, PKN1, F2R, PDGFRA, and HSP90AB1 are downregulated in imatinib- and nilotinib-resistant K562 clones with knockdown of LN892, PROX1-AS1, or SENCR (Fig. 6C). These findings place PI3K signaling as downstream of all three lncRNAs PROX1-AS1, SENCR, and LN892 in resistant cells.

Finally, the merits of the aforementioned genes within PI3K signaling warrant further insights before we can abrogate this signaling and assess the consequences to TKI resistance. We examined the expressions of ITGA2, COL6A1, cyclin D1, PKN1, F2R, PDGFRA and HSP90AB1, and found that these genes are significantly upregulated in imatinib or nilotinib-resistant versus sensitive K562 cells (Fig. 6D) and in TKI-resistant CML patients compared to responding counterparts (Fig. 6E). To address the clinical relevance of these PI3K signaling downstream effectors that we unearthed in vitro, we first compared the expression of ITGA2, COL6A1, cyclin D1, PKN1, F2R, and PDGFRA among patients at a diagnosis with follow-up information available. In AML patients, a trend towards higher levels of expression of most genes was observed in the ELN risk groups intermediate and adverse (Fig. S10A). In line, a trend towards higher expression of COL6A1, F2R, and ITGA2 at diagnosis in AML patients that did not achieve CR after induction chemotherapy was observed (Fig. S10B). In line, a trend towards higher expressions of COL6A1, F2R, and ITGA2 was seen in the blast phase CML compared to samples at initial diagnosis (Fig. S10C). Notably, the predictable capacity is heterogeneous and not robust, which warrants further verification by cofounding with multiple factors, including blast percentage, genetic mutations, age, and sex, in larger cohorts of patients. We then analyzed public datasets (GSE12417 or TCGA-AML) to examine whether the expression of these targets is associated with patient survival. We found that the upregulation of ITGA2, COL6A1, cyclin D1/CCND1, PKN1, PDGFRA, and F2R genes predicts or has trends toward worse outcomes (Fig. 6F). This result was further verified by the findings from GEPIA-mediated analysis (Fig. S10D). Collectively, these results provide compelling evidence that key components of the PI3K signaling are responsible for lncRNA-sustained TKI-resistant cell growth.

### TKI-resistant cells are sensitive to PIK3 inhibitor alpelisib in vitro and in vivo

**Fig. 7:**
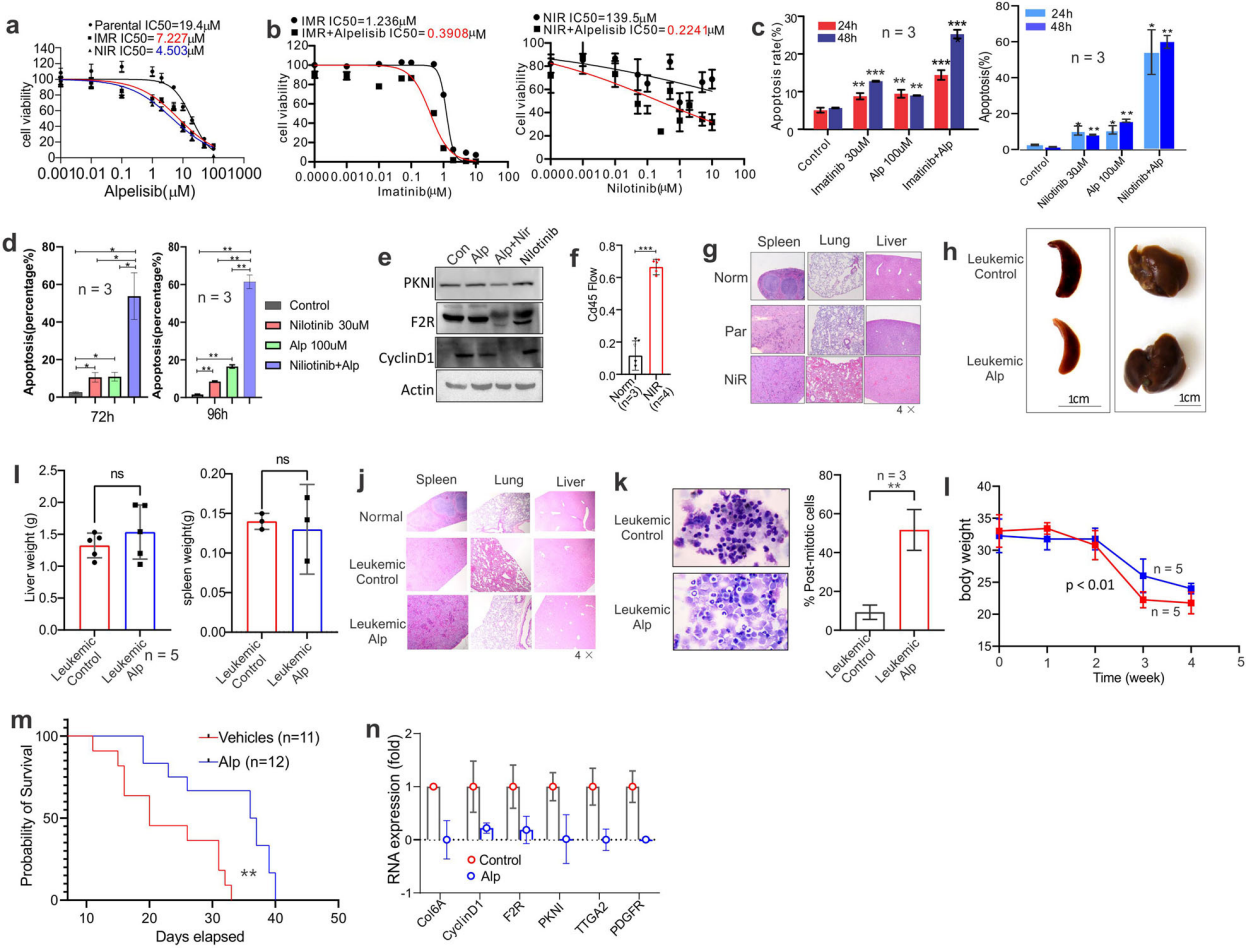
Inactivation of PI3K signaling by alpelisib in TKI-resistant cells achieves leukemia remission in vitro and in vivo. **A** CCK-8 assays in K562 parental and resistant cells to imatinib (IMR) or nilotinib (NIR) with various concentrations of alpelisib for 72 h. **B** CCK-8 assays in K562 IMR or NIR resistant cells with various concentrations of imatinib or nilotinib with/without alpelisib (100 μM) for 72 h. **C**–**E** IMR or NIR resistant K562 were treated with alpelisib (Alp; 100 μM), imatinib (30 μM), or nilotinib (30 μM) alone or in combination for the indicated time points. The treated cells were subjected to flow cytometry (**C**, **D**) for cell apoptosis or Western blot (72 h; **E** Graphs show the quantification of apoptotic cells presented as a percentage. **F**, **G** About 0.5 × 10^6^ nilotinib-resistant K562 cells were injected through the tail-vein into sublethally irradiated NSGS mice (*n* = 5 mice/group). The BM cells were isolated for (**F**) FACS analysis of the engrafted recipient BM cells (stained by CD44 antibody) from the representative disease mice (5 weeks after cell injection). Graphs are the quantification of CD45+ cells (folds). H&E staining of lungs, liver, or spleen sections (×4) from healthy (norm) or leukemic mice bearing parental (Par) or nilotinib-resistant (NIR) cells (**G**). **H**–**N** About 0.5 × 10^6^ BM cells isolated from F were injected via the tail-vein into sublethally irradiated NSGS mice (second recipient). Five days after cell injection, the mice were randomly grouped and treated with alpelisib. Representative external views of the spleen from the leukemic mice (**H**) and the weight of spleen and liver (**I**). **J** H&E staining of lungs, liver, or spleen sections (×4) from healthy (normal), leukemic mice treated with vehicles (control) or alpelisib (*n* = 3) (×4). **K** Representative images of Wright-Giemsa-stained cytospins of BM cells from leukemic mice (left; ×40) and graph (right) show quantification of post-mitotic cells from. **L** Graphs show the changes in body weight of vehicles or alpelisib-treated mice (*n* = 10). **M** Effects of alpelisib on survival of leukemia-bearing mice were determined by the Kaplan–Meier estimate (log-rank test). **N** BM cells isolated from treated mice were subjected to qPCR for the indicated lncRNA targets using primers of human genes. In (**A**, **B**), data represent two independent experiments with eight repeats in total; in (**C**–**E**), the data represent three independent experiments. Note, the survival time is from the start of the leukemia cell injection. Data is shown as means ± SD; Alp alpelisib; *P < 0.05; **P < 0.01; ***P < 0.001.

Having shown that PI3K signaling is essential for leukemia TKI resistance, we reasoned that pharmacological targeting of PI3K signaling could override resistant cells. To test this, we selected alpelisib, an FDA-approved first PI3K inhibitor for breast cancer, which has not been tested in leukemia. When parental or resistant K562 cells were treated with alpelisib in vitro for 72 h, the growth of drug-resistant cells was significantly inhibited in a dose-dependent manner with IC_50_ values in parental cells that are 2.7-fold or 4.3-fold higher than those in IMR or NIR cells (Fig. 7A). The lower IC_50_ values in resistant cells indicated the higher activation of PI3K signaling and thus more sensitivity to alpelisib treatment. Further, the addition of alpelisib lowered the IC_50_ of imatinib by 3.5-fold and the IC_50_ of nilotinib by 625-fold in imatinib- or nilotinib-resistant cells (Fig. 7B). We then treated resistant cells with alpelisib alone or in combination with imatinib or nilotinib for 24, 48, 72, or 96 h. Although alpelisib, imatinib, or nilotinib as single drugs marginally slowed down resistant cell growth, their combination resulted in more pronounced impairment of cell proliferation, as demonstrated by the highest rate of cell apoptosis (Fig. 7C, D). Mechanistically, the combination of alpelisib with nilotinib led to more reduction of PIK3 signaling mediators, such as PKNI, F2R, and Cyclin D1 (Fig. 7E). These results imply that PIK3 signaling is a therapeutic target for overcoming TKI resistance.

To further determine the clinical implications of PI3K signaling in TKI resistance, we established a CML mouse model by injecting 0.5 × 10^6^ nilotinib-resistant K562 cells via the tail-vein into sublethally irradiated (2.5 Gy) triple transgenic NSG-SGM3 (NSGS) mice (male). The successful engraftment of CML cells was verified by the identification of human CD45+ cells in mouse BM cells (Fig. 7F) and the infiltration of CML K562 cells into mouse organs (e.g., liver, lung, spleen) (Fig. 7G). Then the BM cells (0.5 × 10^6^) were isolated from mice bearing K562 resistant cells and injected into the second recipient NSGS mice 4 h after sublethal irradiation. These leukemic mice were randomly grouped and sequentially given 30 or 50 mg/kg of alpelisib [72] in PEG400 and saline (ratio 15:38:47) intraperitoneally twice a week for a total of eight doses. The leukemic mice injected with only the vehicle served as controls. Although the size and weight of the spleen and liver did not show a significant difference (Fig. 7H, I), H&E staining revealed that, compared to the alpelisib-treated mice, the vehicle group displayed an increased infiltration of leukemic cells into the spleens, lungs, and livers of recipients, leading to considerable damage to these organs (Fig. 7J). The BM histopathology from alpelisib-treated mice identified more differentiated cells containing metamyelocytes, bands, and segmented neutrophils compared to vehicle-treated mice (Fig. 7K). Alpelisib treatment significantly slowed the decrease in body weight (Fig. 7l) and importantly, increased the survival time of leukemic mice (Fig. 7M). No toxicity was observed for the tested drug dosage and schedule because we did not see any evident change in the capability of moving and getting food and water when comparing alpelisib-treated mice to vehicle groups. Mechanistically, alpelisib administration in mice remarkably downregulated PI3K signaling mediators, including ITGA2, COL6A1, cyclin D1/CCND1, PKN1, PDGFRA or F2R (Fig. 7N). Of note, the leukemic mice were bearing human CML K562 cells; thus, we used primers for human, but not mouse, genes to detect the above genes. This approach could verify the limited toxicity of alpelisib to leukemic mice and demonstrate the specificity of CML cell killing by alpelisib in vivo. Together, these findings provide critical means of targeting PI3K signaling to treat patients with refractory leukemia post TKI therapy.

## Discussion

Treatment with TKIs often leads to the development of resistance, which has been a major hurdle to successful cancer treatment. However, mechanisms that generate and sustain drug-resistant cell populations, as well as molecular predictors for prognosis and drug response, are still unclear, particularly for the resistant patient subpopulation without acquired TK kinase domain mutations. By dissecting the impact of a dynamic m^6^A methylome on TKI sensitivity, we demonstrate that the m^6^A-regulated lncRNAs are deregulated molecules whose overexpression in TKI-resistant cell lines and in patients with inadequate response to TKI therapy is important not only for resistant cell proliferation, but also for insensitivity to TKI treatment and patient survival. We also show that the FTO-dependent m^6^A demethylation can regulate lncRNAs by increasing their RNA stability, and lncRNA-activated PI3K signaling renders insensitivity to TKIs and sustains resistant cell growth. Further, we observe that, among the refractory or relapsed population, FTO levels are significantly higher in patients without kinase mutations compared to those with mutations; lncRNA upregulation and the activated PI3K signaling detected in patients at diagnosis are associated with the follow-up bad drug response or unfavorable outcomes. Our findings add a new layer to the complexity of mechanisms regulating leukemia cell fate under TKI selection, and raise the possibility that the m^6^A-regulated lncRNAs represents a new non-genetic factor to affect the development and maintenance of TKI resistance; our discoveries identify a promising therapeutic target, FTO, for specifically, the most challenging patient subpopulations who are TKI non-responders/relapsed but do not carry the acquired BCR::ABL1 mutations; our results uncover a strong predictor, m^6^A-regulated lncRNA-PI3K axis, for poorer prognosis and failure in drug response in cancers.

Acquired resistance to TKIs is commonly attributed to genetic mechanisms [8, 73]. However, compelling evidence supports that the development of drug resistance is accomplished without genetic alterations [74–76]. Targeting epigenetic machinery has become a major thrust in the development of new therapeutic strategies; unfortunately, these strategies have yielded mixed results in clinical trials. One barrier to progress in harnessing epigenetic therapies to overcome drug resistance is the limited understanding of how cancer cells can rapidly escape TKI killing. We recently demonstrated [37] that a dynamic and reversible m^6^A methylome represents a non-genetic driver of a TKI-tolerance state in heterogeneous leukemia cells, yet the molecular mechanisms by which the TKI-altered m^6^A methylome affects drug sensitivity are incompletely understood. Because transcriptome-wide m^6^A sequencing disclosed that most changes of m^6^A peaks in resistant cells come from lncRNAs [37], this study was designed to test the hypothesis that m^6^A-associated lncRNAs could be an additional key non-genetic factor to fuel and sustain resistant cell growth. As expected, we identified and validated many annotated lncRNAs, which are differentially expressed with an obvious change of m^6^A content in TKI-resistant cell lines and non-responding leukemia patients.

LncRNA defects have been found to affect drug resistance [77], but it is unclear whether an interaction between m^6^A and lncRNAs exists in leukemia TKI resistance. To test if m^6^A-associated lncRNAs play a role in TKI response, we initially examined their role in leukemia patients. We observed that these lncRNAs, including PROX1-AS1, SENCR, or LN892, are elevated in leukemia patients vs normal donors, and in CML patients not responding to TKIs or with blast crisis disease compared with TKI responders or with chronic disease. We further observed that patients with higher lncRNA expression at least have a trend toward worse prognosis than those with lower levels. These findings support PROX1-AS1, SENCR, or LN892 as prognostic biomarkers in leukemia therapy. We next knocked down the upregulated PROX1-AS1, SENCR, or LN892 in resistant cells, and found that lncRNA knockdown inhibits resistant cell growth and sensitizes nilotinib-resistant cells to nilotinib killing. These findings suggest that lncRNA upregulation is required, at least partially, to support resistant cell growth and to preserve TKI insensitivity.

It is increasingly appreciated that lncRNAs are key regulators in cancer pathogenesis and drug resistance [39–47]. However, they are not the direct contributors but determine cancer cell fate through up- or down-regulating cancer cell determinant genes. Further, very few studies have been found to address the role of PROX1-AS1, SENCR, or LN892 in drug resistance. By analyzing a gene expression profile resulting from SENCR knockdown, we identified a downstream mediator, PI3K signaling, which represents a central regulatory node controlling cell proliferation and apoptosis, and plays a critical role in leukemia drug resistance [78]. The key genes (i.e., ITGA2, COL6A1, cyclin D1, PKN1, PDGFRA, F2R), which are changed the most and likely involved in the PI3K signaling, could be downstream targets of PROX1-AS1, SENCR, or LN892, because knockdown of these lncRNAs impairs the above target expression. These lncRNA-regulated genes are overexpressed in TKI-resistant cells and in imatinib or nilotinib non-responding patients compared to good responders. Leukemia patients with overexpression of ITGA2, COL6A1, cyclin D1, PKN1, PDGFRA, or F2R predict or have trends toward worse prognosis. However, it will be interesting to see in the future whether these PI3K targets are prognostic biomarkers in larger cohort of patients, which should also be justified by age, mutation status, sex, disease stages and induction pre-treatment; moreover, the clinical implications of key components within PI3K pathway identified in GSE51878 warrant further investigations in RNA sequencing data generated from relevant AML or CML models with knockdown of PROX1-AS1, SENCR, or LN892.

The molecular basis of lncRNA dysregulation in resistant leukemia is still obscure. We focused on m^6^A methylation in regulating lncRNAs, because (1) a dynamic m^6^A methylome represents a bona fide defense mechanism in developing TKI resistance; (2) half of the differentially expressed lncRNAs have m^6^A motifs; (3) m^6^A reduction and lncRNA upregulation co-exist in resistant cells; (4) the abundance of m^6^A plays a key role in RNA stability; and (5) the transcriptional repression by lncRNAs is enhanced by m^6^A methylation [48, 49]. Our studies revealed that, in parallel with global m^6^A hypomethylation, m^6^A content specific for upregulated lncRNAs, like PROX1-AS1, SENCR, or LN892, is significantly decreased in resistant cells. Given that upregulation of these lncRNAs affects TKI sensitivity and patient survival, these results support a crosstalk between the m^6^A methylome and the lncRNA pathway in TKI resistance. Mechanistically, FTO serves as a new lncRNA regulator through FTO-dependent m^6^A hypomethylation in leukemia-resistant cells. In support of this possibility was the downregulation of PROX1-AS1, SENCR, or LN892 and the reduction of their m^6^A levels when FTO is inactivated by either gene knockdown or inhibitor treatment. This is followed by an increased RNA half-life of lncRNA transcripts. These findings provide an alternative explanation for why many lncRNAs are differentially expressed in TKI-resistant cells. It would also be important to know whether all identified lncRNAs are direct targets of FTO and whether FTO-dependent m^6^A affects RNA stability of all these targets.

While TKIs (e.g., imatinib, nilotinib) have revolutionized leukemia treatment [2–5], TKIs don’t cure all leukemia patients, due to the development of TKI resistance. Further, a subpopulation of patients (30–50% in CML) who achieve complete molecular remission must take TKIs for the rest of their lives, which can lead to severe side effects and substantial financial burden. Thus, the development of novel therapies to overcome resistance is a major goal in the field. After demonstrating the key role of the lncRNA-PI3K axis in regulating TKI resistance, we explored the therapeutic potential of the PI3K inhibitor alpelisib in treating patients with resistant CML. Notably, PI3K signaling is an important downstream mediator in BCR-ABL-driven leukemia, but alpelisib has not been tested, particularly, for refractory CML patients. We showed that alpelisib sensitizes imatinib or nilotinib-resistant cells to imatinib or nilotinib-induced cell death, and its combination with imatinib or nilotinib results in more pronounced cell apoptosis in vitro. Most importantly, alpelisib therapy in NSGS mice bearing nilotinib-resistant K562 cells significantly reduces leukemia burden and increases the survival time of leukemic mice, mechanistically through suppression of PI3K signaling mediators like F2R, Cyclin D1, and PDGFR. These findings support alpelisib as a novel therapeutic reagent for TKI-resistant leukemia, opening promising new avenues to treat patients with recurrent diseases. Future studies will perform an lncRNA-related rescue experiment in vitro and in vivo to directly address the functional relationship between the lncRNAs and the PI3K pathway. Findings will substantiate the mechanistic basis of the lncRNA-activated PI3K pathway in leukemia TKI resistance.

In summary, this study discovers a critical role that m^6^A-regulated lncRNAs play in sustaining resistant growth, maintaining resistant phenotypes, and promoting TKI insensitivity in vitro and in CML patients receiving TKI therapy. It reveals a novel mechanism, the FTO-m^6^A-lncRNAs axis, to explain why lncRNAs are elevated in TKI-resistant cells, and a pathway, lncRNA-regulated PI3K signaling, to answer the question of how lncRNA dysregulation affects TKI resistance. Given that m^6^A-regulated lncRNAs affect TKI response and survival of leukemia patients, it will be interesting to see in the future whether these hitherto underappreciated roles for m^6^A-lncRNAs-PI3K cascade in anti-cancer resistance could serve as a foundation to design therapeutics that might overcome TKI resistance for this devastating cancer.

### Limitations of the study

The study has some limitations. First, we revealed a dynamic RNA m^6^A methylome [37] and identified a set of lncRNAs (see Fig. 3), which are shared by AML and CML cells post TKI treatment. The current study used only CML as a readout model to get further insights into cancer drug resistance. The role of m^6^A-regulated lncRNAs in AML might be challenging to explore due to the “7 + 3” regimen before TKI therapies. Second, the identification and characterization of lncRNA abnormalities were limited to relatively small cohorts of patients at the diagnostic stage, blast crisis phase, or TKI non-responders. The use of the tested lncRNAs as molecular predictors for drug resistance and poorer prognosis needs to be validated in larger cohorts of patients from multiple centers. Third, our animal models to pharmacologically test inhibitors (e.g., alpelisib) for the lncRNA-PI3K axis are limited to leukemic mice bearing resistant cell lines. The patient-derived xenograft models bearing nilotinib-resistant CML patient blasts can further explore the clinical implications of alpelisib in overcoming leukemia TKI resistance. Fourth, we only targeted PI3K signaling, one of multiple pathways controlled by dysregulated lncRNAs in resistant cells, to overcome TKI resistance. Given the key roles of high m^6^A-enriched lncRNA expression in resistant patient cells, future studies can further exploit the therapeutic potentials of targeting lncRNAs themselves (e.g., SENCR, PROX1-AS1, LN892), which are more challenging due to in vivo delivery approaches but with higher efficacy due to multiple downstream effector pathways. Fifth, by our knowledge, GAPDH is not subjected to regulation by RNA m^6^A. Thus, the levels of GAPDH in m^6^A IP may relatively reflect the total RNA quantity, which is suitable to be an internal control to normalize for variations in the efficiency of the m^6^A IP process across samples. However, future studies should also include inputs as internal controls, which would be beneficial for more precise quantification of m^6^A enrichment and improve the ability to distinguish subtle changes in m^6^A from changes in target expression.

## Supplementary information

Supplementary Materials-Clean

Uncropped gel images

Suppl Table 1

Suppl Table 2

Supplementary Tables 4 and 5

## Data Availability

All relevant data supporting the key findings of this study were available within the article and its Supplementary Information files or from the corresponding authors upon reasonable request. Partial data were obtained from “Public data analysis,” including GEO datasets (GSE80481; GSE51878).
