# Supplementary material for: The RNA N6-methyladenosine methylome coordinates long non-coding RNAs to mediate cancer drug resistance by activating PI3K signaling
Source: Cell Death Dis. 2025 Nov 7;16(1):804. doi: 10.1038/s41419-025-08045-6 (PMC12594950; doi:10.1038/s41419-025-08045-6)
Supplement: Supplementary file 1 — Supplementary Materials-Clean [file 41419_2025_8045_MOESM1_ESM.docx]

**Supplementary Information**

**The RNA N⁶-Methyladenosine Methylome Coordinates Long Non-Coding RNAs to Mediate Cancer Drug Resistance by Activating PI3K Signaling**

Yanhong Tan, Changli Zhou, Sicheng Bian, Huiqin Bian, Yanan Ren, Wencke Walter, Jiuxia Pang, Tao Cheng, Hongwei Wang, Yuchao Yang, Wenzheng Guo, Lingli Zhang, Aref Al-Kali, Mark R. Litzow, Xiaonan Han, Jianhua Yu, Rendong Yang, Gang Huang, Gregor Hoermann, William Tse, Shujun Liu

**Inventory of Supplemental Information**

**Supplemental Figures and Legends**

- **Figure S1, Related to Figure 1**
- **Figure S2, Related to Figure 2**
- **Figure S3, Related to Figure 2**
- **Figure S4, Related to Figure 3**
- **Figure S5, Related to Figure 3**
- **Figure S6, Related to Figure 3**
- **Figure S7, Related to Figure 4**
- **Figure S8, Related to Figure 5**
- **Figure S9, Related to Figure 6**
- **Figure S10, Related to Figure 6**

**Supplemental Tables**


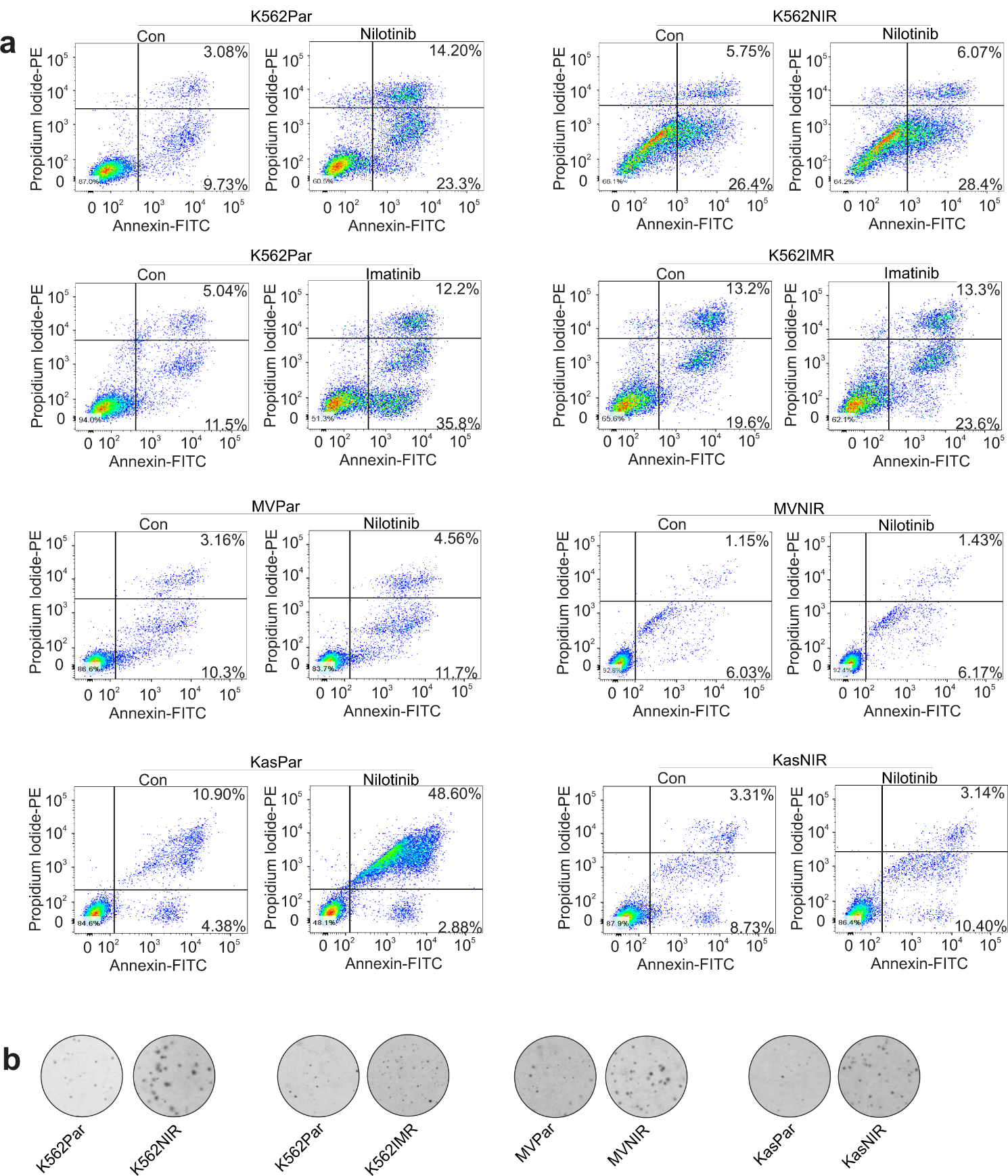


**Figure S1. Characterization of TKI resistant cells. (a)** Flow cytometry assays in parental and resistant cells treated with 1 µM nilotinib or imatinib for 72 hours. Data represents three independent experiments. **(b)** Colony forming assays for parental and resistant cells. Data represents two independent experiments with three duplicates. Par, parental control; Con, control; NIR, nilotinib resistance; IMR, imatinib resistance; Kas, Kasumi-1; MV, MV4-11.

 **a**

**b**

**Figure S2. Analysis of lncRNA-associated m^6^A peak and account**. (**a**) Venn diagram illustrating the number of overlapped lncRNAs highly expressed in resistant or parental cells with m^6^A-associated lncRNAs. (**b**) qPCR of total RNA for the expression of indicated lncRNAs in parental and resistant K562 cells. P, parental control; NR, nilotinib resistance; IR, imatinib resistance.


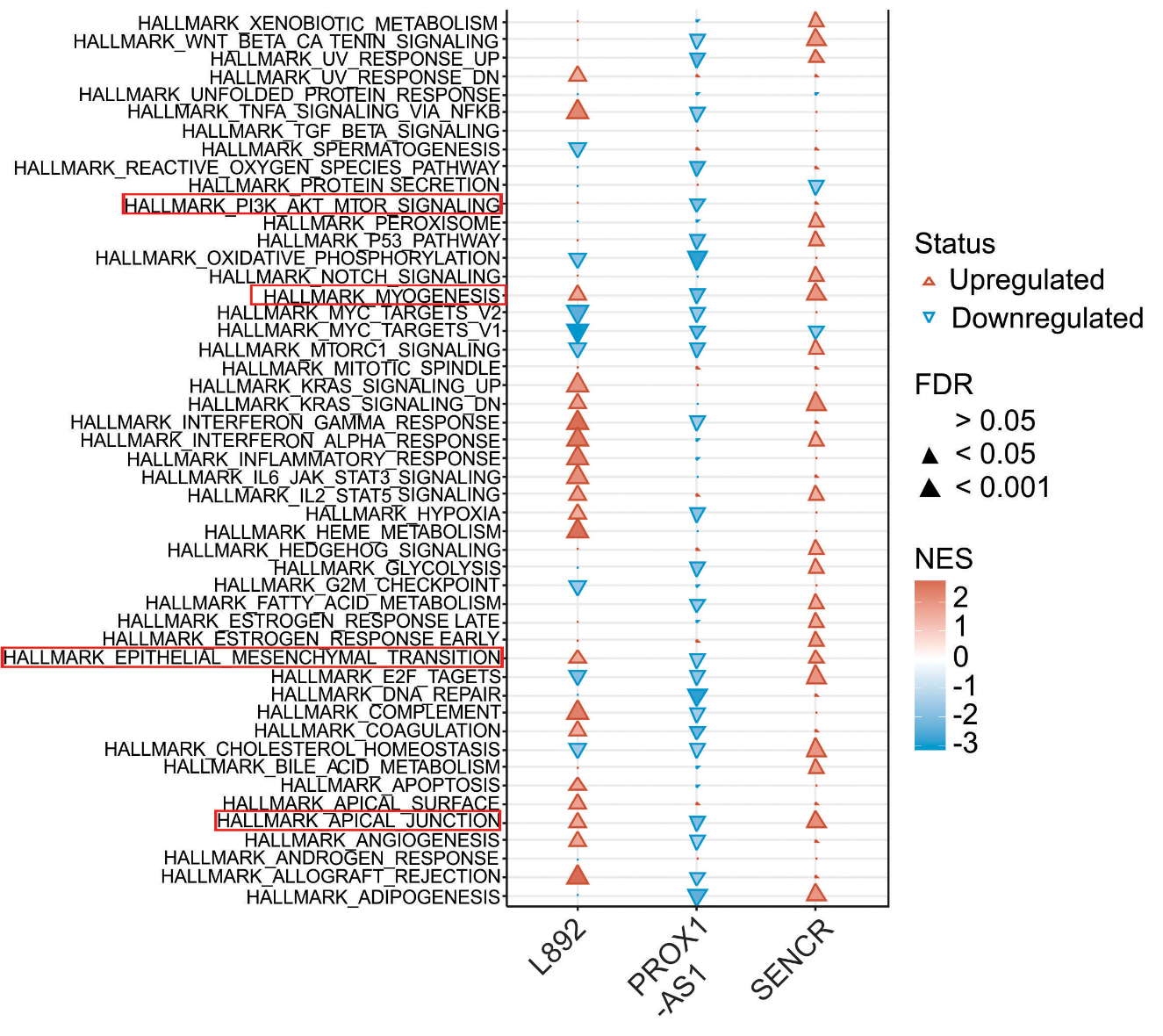
**Figure S3. Pathways associated with the upregulated lncRNAs**. The correlation of expression between each lncRNA and other genes is used for GSEA pre-rank analysis. Hallmark gene sets are used for pathway analysis; NES stands for Normalized Enrichment Score, the higher the score, the more positive enriched in that pathway; the lower the score, the more negative enriched; FDR ≤ 0.05, pathways are significantly enriched; FDR > 0.05, not significantly enriched.


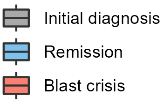

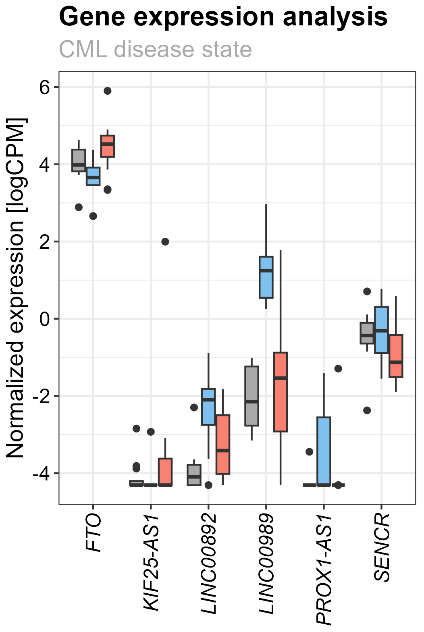

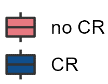

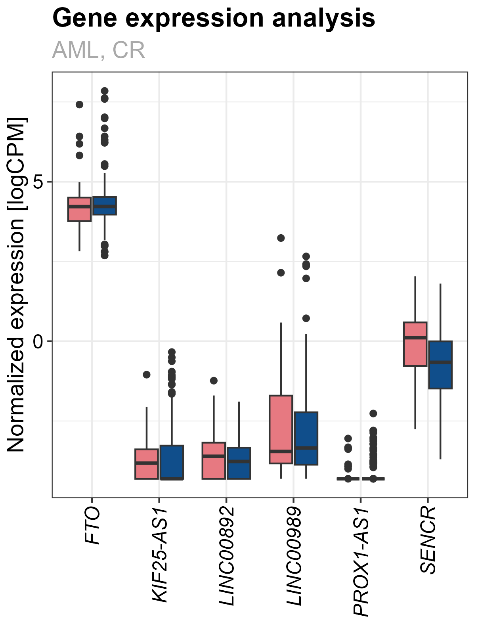

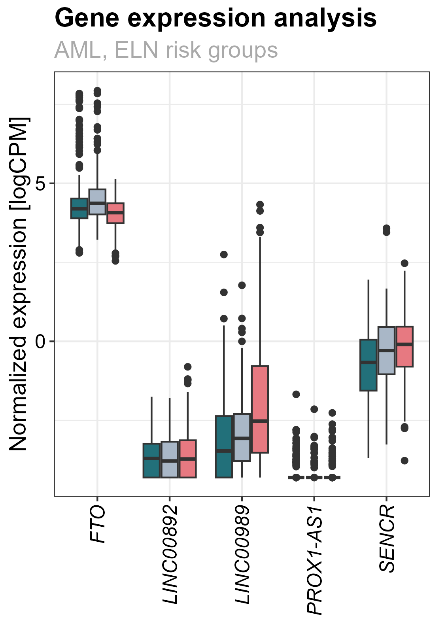
**a b c**


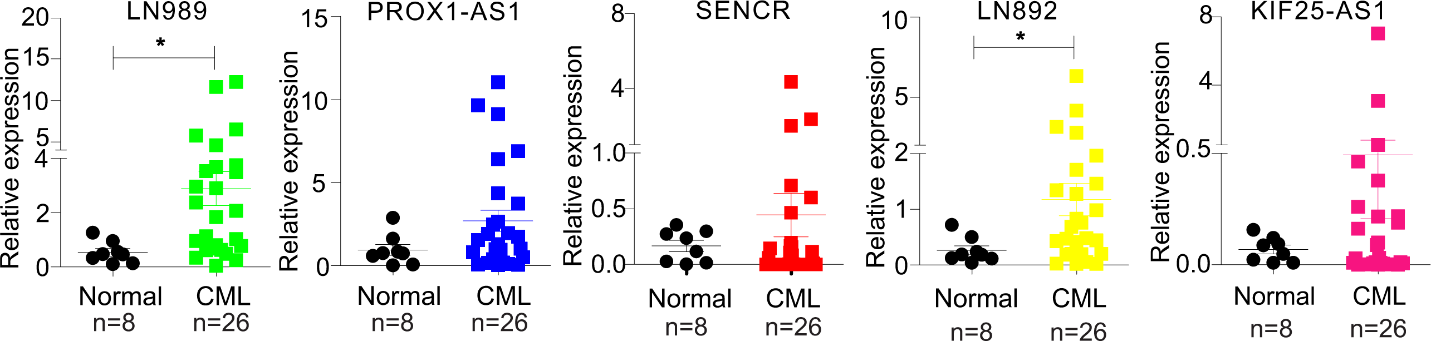

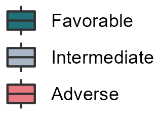
**d**

**Figure S4. Expression of FTO and lncRNAs in AML and CML patients.** (**a**, **b)**, Analysis of gene expression profiling in patient cells collected at diagnosis. AML patients (n = 774) were stratified according to ELN risk group (**a**) and the achievement of complete remission after induction therapy (**b**). (**c**) Matched samples from CML patients (n = 12) were analyzed at initial diagnosis of chronic phase CML, in hematologic remission, and at progression to blast phase CML. (**d**) qPCR for lncRNA expression in CML patients (n = 26) and normal donors (n = 8). **P* < 0.05.


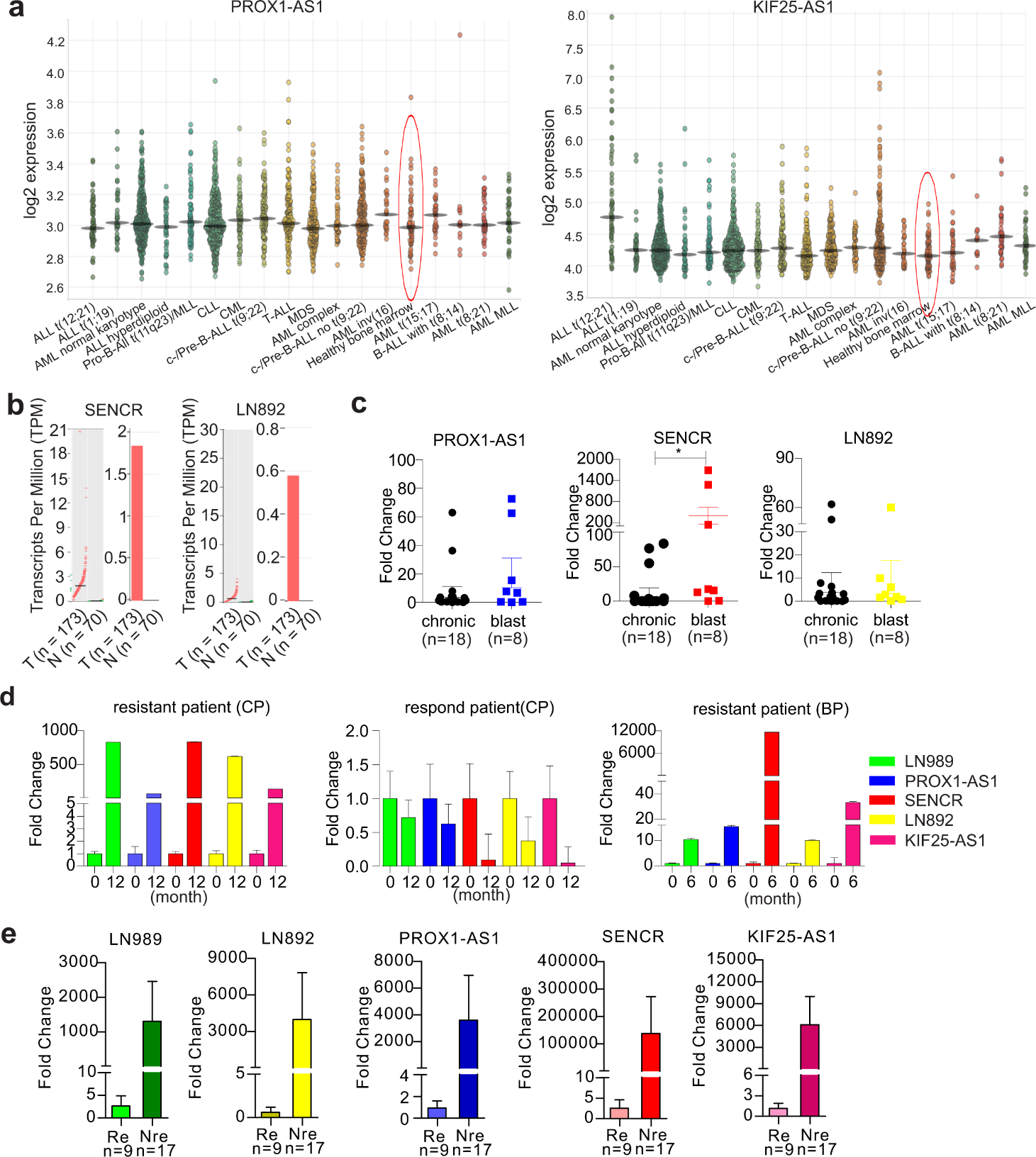


**Figure S5.** **LncRNAs are upregulated in leukemia patients.** (**a**,**b**) Public dataset analysis for the expression of indicated lncRNAs using online tools BloodSpot (Leukemia MILE Study) (**a**) or GEPIA (**b**). The images were generated by these tools. The red circles in (**a**) indicate lncRNA expression in normal donors. Note, in GEPIA analysis, Dot plot: The gene expression profile across all tumor samples and paired normal tissues; each dot represents expression of samples. Bar plot: The gene expression profile across all tumor samples and paired normal tissues. The height of bar represents the median expression of certain tumor type or normal tissue. (**c**) Expression of PROX1-AS1, SENCR and LN892 in chronic (n = 18) and blast crisis (n = 8) CML patients using ABL as an internal control. (**d**) Expression of lncRNAs in CML-CP patients with TKI respondence and resistance, respectively, and in individual CML-BP patients with TKI resistance. (**e**) qPCR for lncRNA expression of LN989, LN892, PROX1-AS1, SENCR and KIF25-AS1 in responder (n = 9) and inadequate responder (n = 17) CML patients using 18S as an internal control. T, tumor; N, normal; Re, responder; Nre, non-responder; CP, chronic phase; BP, blast phase.


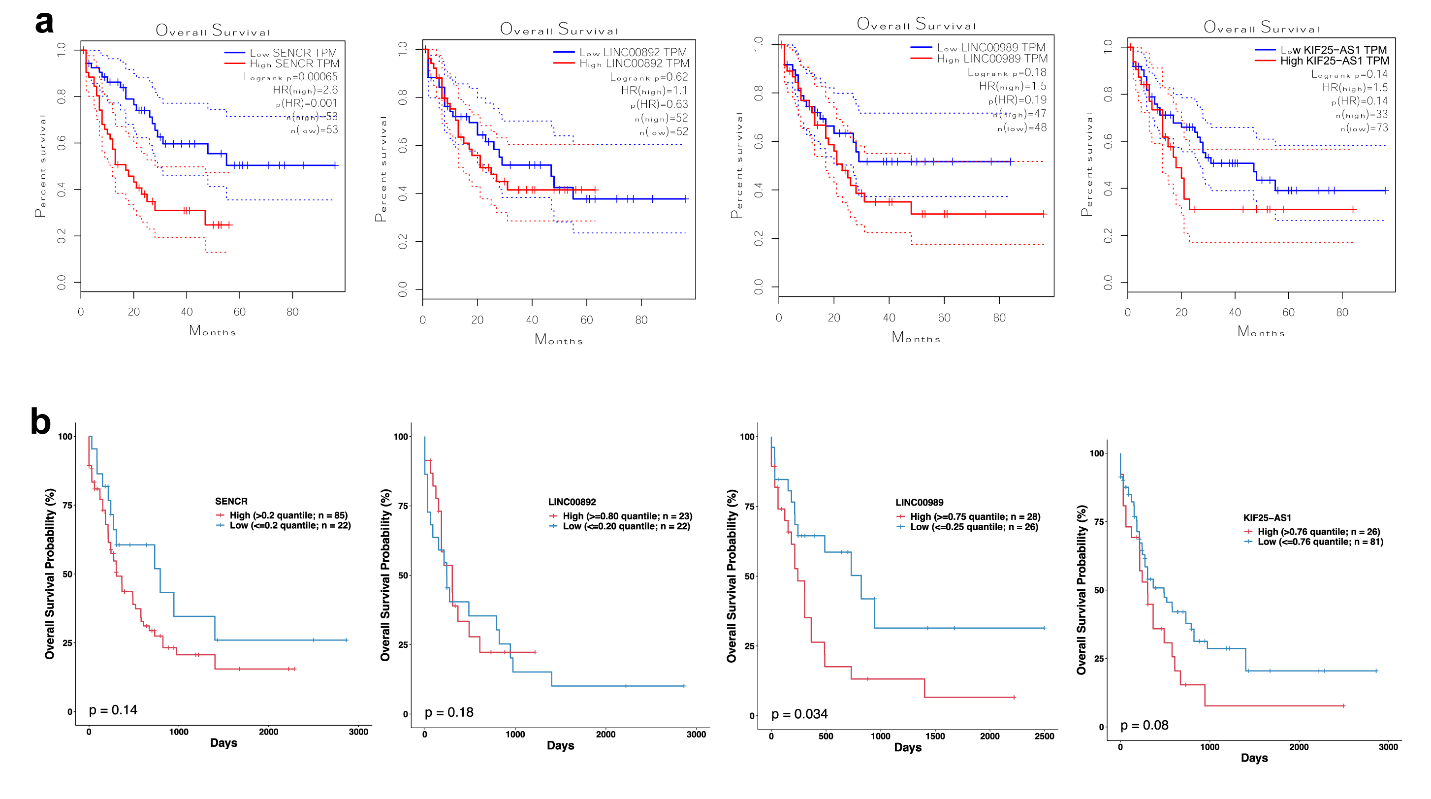


**Figure S6. Upregulation of lncRNAs predicts unfavorable outcomes in AML patients.** The association of lncRNA expression with overall survival (OS) in TCGA LAML (leukemia) dataset analyzed by the Kaplan–Meier estimate (n = 200). (**a**) The plots were made using a database which provides LAML expression and clinical information for analysis. (**b**) The plots were generated using TCGA LAML data. Log-rank test; the quantiles/percentiles were indicated in the plots.


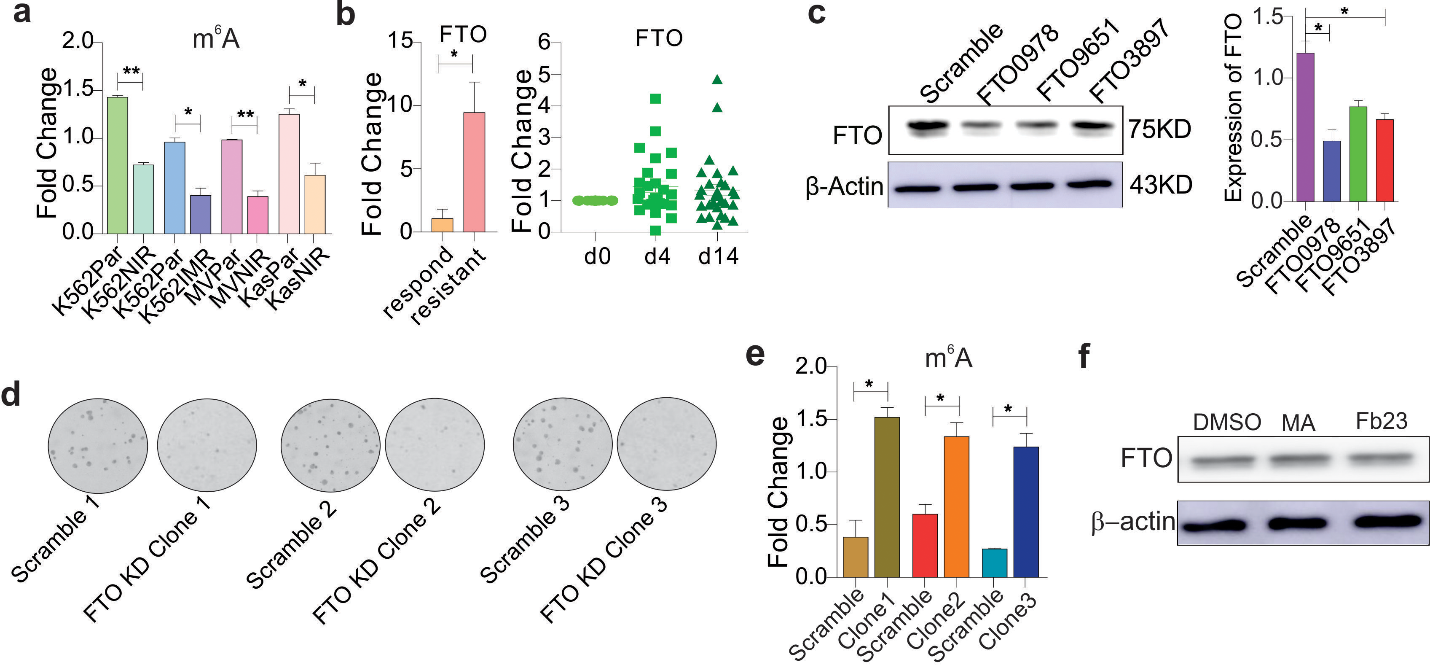


**Figure S7**. **FTO regulates lncRNA expression.** (**a**) Quantification of dotblotting reported in Fig. 4A. (**b**) qPCR for FTO expression in CML (n = 16) and AML (n = 32) patients receiving nilotinib or imatinib therapy. (**c**) K562 parental cells were infected by different FTO shRNA viruses and selected by 1 µg/ml puromycin for 5 days. Western blot was used to measure FTO protein expression. Graph shows the quantification of Western blot band intensities. (**d**) K562 resistant cells were infected by FTO shRNA virus TRCN0000180978 and subjected to colony-forming assays. Data represents two independent experiments with three duplicates. (**e**) Quantification of dotblotting reported in Fig. 4E. (**f**) Western blotting for FTO protein expression in K562 resistant cells treated with FTO inhibitors for 6 hours. Data represents 3 independent experiments. **P* < 0.05, ***P* < 0.01.


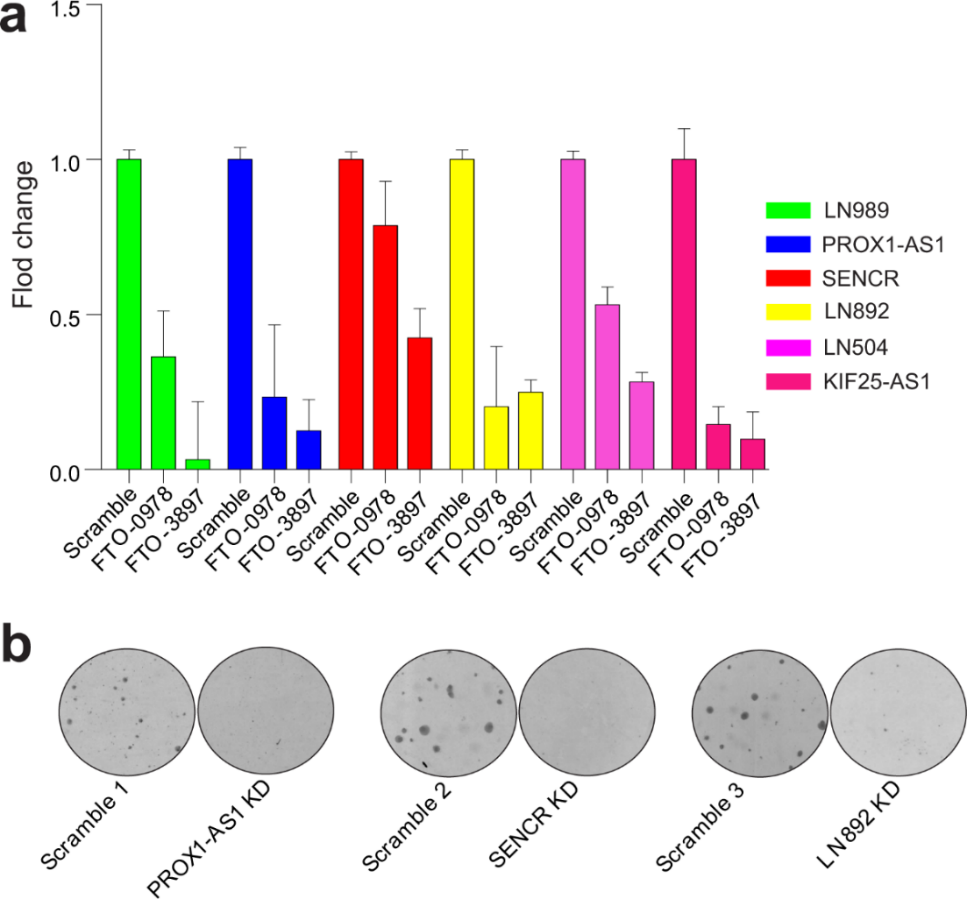


**Figure S8.** **The effects of FTO knockdown in K562 parental cells.** (**a**) qPCR for lncRNA expression in K562 parental cells with FTO knockdown by 2 shRNA virus infection. (**b**) The colony-forming assays in K562 nilotinib resistant clones with PROX1-AS1, SENCR and LN892 knockdown. In colony assays, data represents two independent experiments with four duplicates.


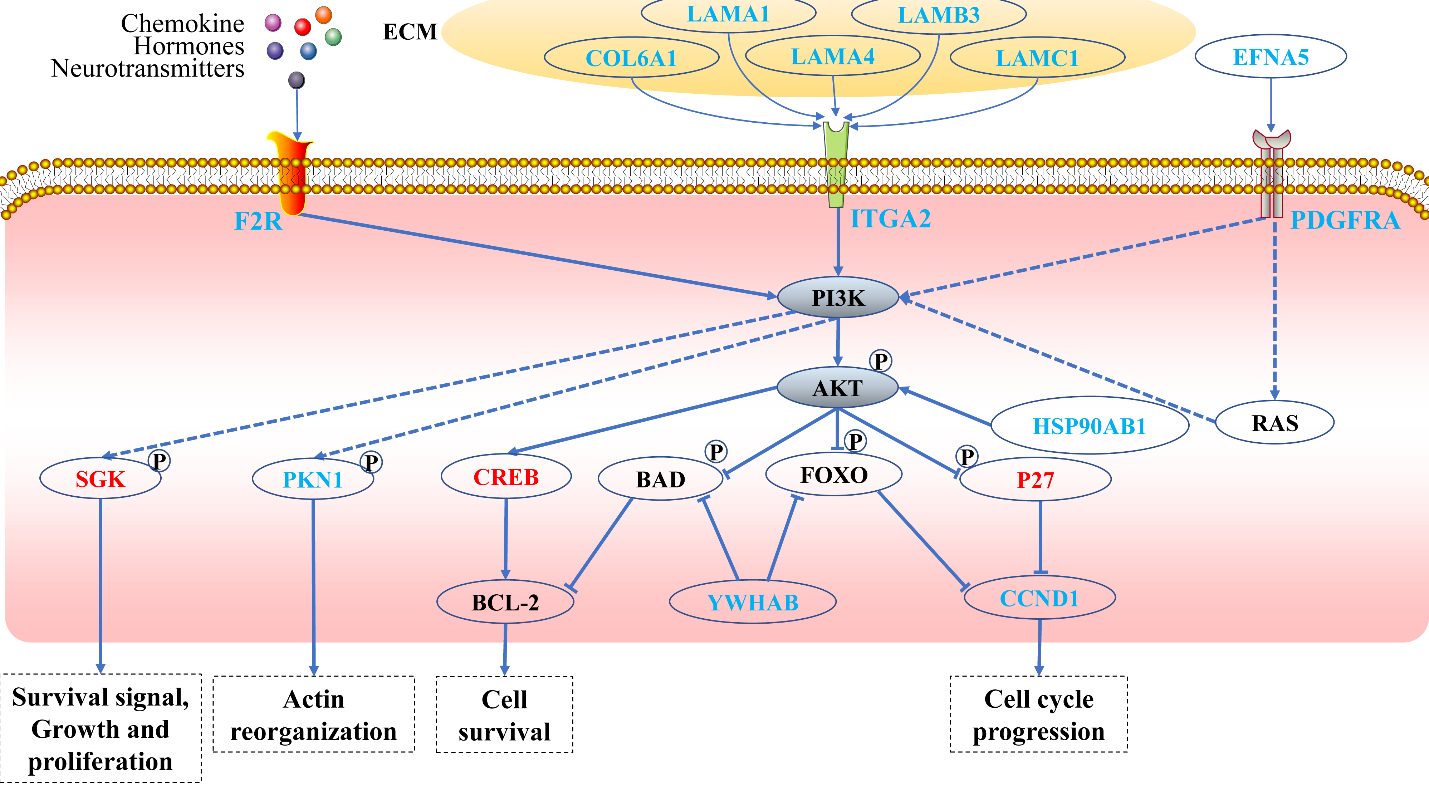


**Figure S9. The PI3K pathway network.** Using DAVID 6.8 for functional annotation, KEGG data for differentially expressed genes in SENCR-knockdown cells identified the PI3K as a major downstream effector pathway. Red genes are the significantly up-regulated genes in SENCR-knockdown cells, the blue genes are the significantly down-regulation genes in SENCR-knockdown cells.


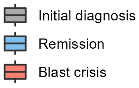

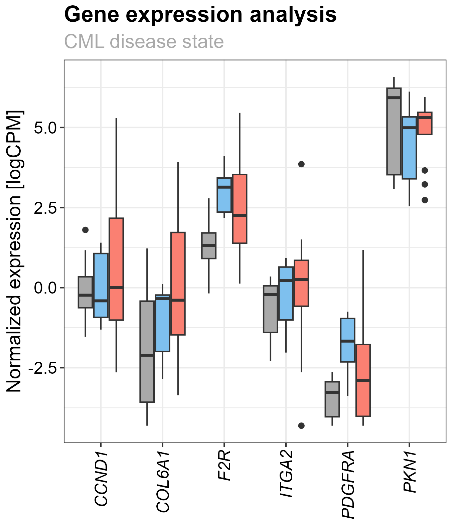

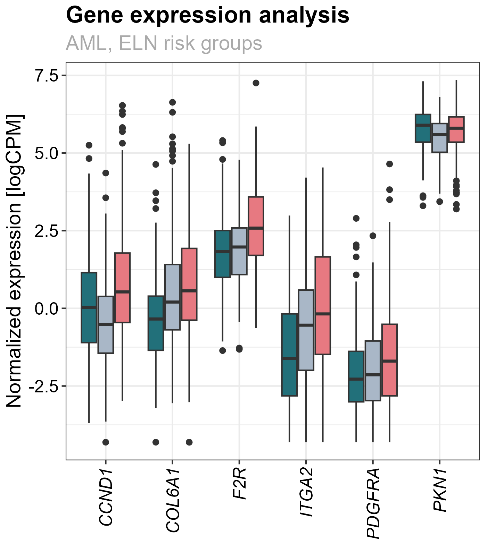
**a b c**


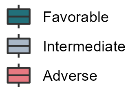

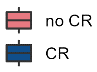

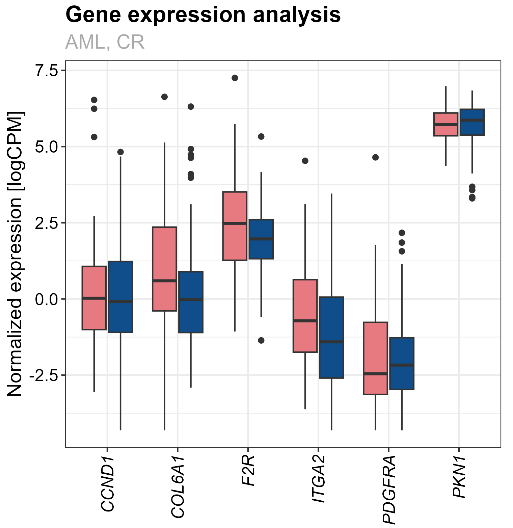

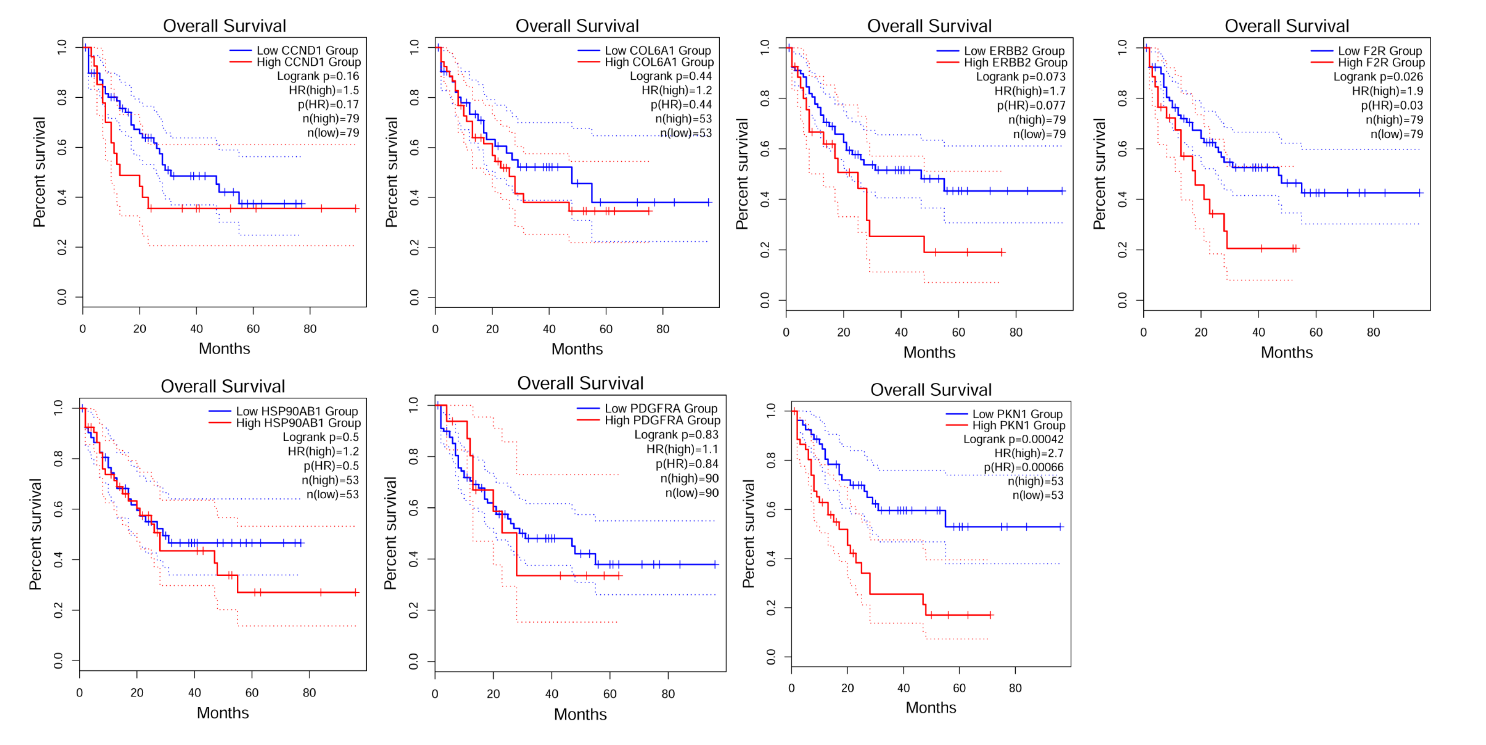
**d**

**Figure S10. Upregulation of PI3K downstream mediators predicts unfavorable outcomes in leukemia patients at a diagnosis and after induction of TKI therapies.** (**a**, **b**) Analysis of gene expression profiling in patient cells collected at diagnosis. AML patients (n = 774) were stratified according to ELN risk group (**a**) and the achievement of complete remission after induction therapy (**b**). (**c**) Matched samples from CML patients (n = 12) were analyzed at initial diagnosis of chronic phase CML, in hematologic remission, and at progression to blast phase CML. (**d**) The association between the expression of PI3K downstream mediators with overall survival (OS) in public dataset analyzed by the Kaplan–Meier estimate using online tool GEPIA.

**Supplementary Table 1.** Differentially expressed lncRNAs in resistant vs. parental K562 cells.

**Supplementary Table 2.** Up- and down-regulated lncRNAs that are associated with m^6^A peaks. DElncRNAs_resistance_m^6^A is a subset of DElncRNAs_resistance that overlapped lncRNAs with m^6^A peaks in resistance file); DElncRNAs_parental_m^6^A is a subset of DElncRNAs_parental that overlapped lncRNAs with m^6^A peaks in parental file.

**Supplementary Table 3** Characteristics of 40 CML patients with TKI treatment.

**Supplementary Table 4** Up- and down-regulated genes in SENCR knockdown cells.

**Supplementary Table 5** Lists of functional pathways identified by DAVID 6.8 for functional annotation. Functional pathways that involve ≥9 genes and *P* value <0.05 are listed.

**Supplementary Table 6 Lists of antibodies used in the experiments**

**Supplementary Table 7 Sequence of primers used in the experiments**

**Supplementary Table 3 Characteristics of 40 CML patients with TKI treatment**

| **Variables** | | **Good response**  **(n = 15)** | **Inadequate response**  **(n = 25)** |
| --- | --- | --- | --- |
| Age (years) | |  |  |
| Median | | 46 | 39 |
| Range | | 15-65 | 15-69 |
| Sex | |  |  |
| Female (N,%) | | 10 (66.7% ) | 10 (40.0%) |
| Male (N,%) | | 5 (33.3%) | 15 (60.0%) |
| Phase | |  |  |
| Chronic phase (N,%) | | 15 (100%) | 16 (62.5%) |
| Blast phase (N,%) | | 0 (0.0%) | 9 (37.5%) |
|  | |  |  |
| WBC (× 10^9^/L) | | 178.0 | 210.6 |
| PLT (× 10^9^/L) | | 534.3 | 373.3 |
| Hb (g/L) | | 104.7 | 112.8 |
| Bone marrow blasts (%) | | 2.1 | 1.7 |
| *BCR::ABL1* transcripts | |  |  |
| *BCR::ABL1 e14a2 or e13/a2 (P210) transcripts* (N, %) | 14 (93.3%) | 24 (96.0%) |  |
| *BCR::ABL1 e1a2 (P190) transcripts* (N, %) | 0 (0.0%) | 0 (0.0%) |  |
| *BCR::ABL1 e14a2 or e13/a2 (P210) transcripts* + *BCR::ABL1 e1a2 (P190) transcripts* (N, %) | | 1 (6.7%) | 1 (4.0%) |
| Chromosome | |  |  |
| Ph (N, %) | | 15 (100%) | 24 (96.0%) |
| ACA (N, %) | | 0 (0.0%) | 1 (4.0%) |
| TKIs | |  |  |
| Imatinib (N, %) | | 7 (46.7% ) | 4 (16.0%) |
| Nilotinib (N, %) | | 3 (20.0% ) | 10 (40.0%) |
| Imatinib + Nilotinib (N, %) | | 5 (33.3% ) | 11 (44.0%) |
| ABL mutation | |  |  |
| T315I (N, %) | | 0 (0.0%) | 2 (8.3%) |
| Others (N, %) | | 0 (0.0%) | 2 (8.3%) |

**Supplementary Table 6 Antibodies used in the experiments**

| Antibody | Application | Company | Catalog No. | Source | Dilution | |
| --- | --- | --- | --- | --- | --- | --- |
| FTO | Western blot | AdipoGen | AG-20A-0064 | Mouse | 1:2000 |  |
| STAT5 | Western blot | Cell Signaling | 9363 | Rabbit | 1:1000 |  |
| p-STAT5 | Western blot | Cell Signaling | 9351 | Rabbit | 1:1000 |  |
| KIT | Western blot | Cell Signaling | 3074 | Rabbit | 1:500 |  |
| p-KIT | Western blot | Cell Signaling | 3391 | Rabbit | 1:500 |  |
| FLT3 | Western blot | Cell Signaling | 3462 | Rabbit | 1:500 |  |
| p-FLT3 | Western blot | Cell Signaling | 3464 | Rabbit | 1:500 |  |
| BCR | Western blot | Cell Signaling | 3902 | Rabbit | 1:500 |  |
| p-ABL | Western blot | Cell Signaling | 2865 | Rabbit | 1:500 |  |
| WTAP | Western blot | Abcam | ab195380 | Rabbit | 1:2000 |  |
| PKN1 | Western blot | Santa Cruz | Sc-393344 | Mouse | 1:1000 |  |
| F2R | Western blot | Thermo Fisher | 35-2200 | Mouse | 1:1000 |  |
| cyclin D1 | Western blot | abcom | Ab16663 | Rabbit | 1:1000 |  |
| β-actin | Western blot | Santa Cruz | sc-47778 | Mouse | 1:1000 |  |
| goat anti-rabbit IgG | Western blot | Cell Signaling | 7074S |  | 1:10000 |  |
| horse anti-mouse IgG | Western blot | Cell Signaling | 7076S |  | 1:10000 |  |
| rabbit anti-goat IgG | Western blot | Invitrogen | 31402 |  | 1:10000 |  |
| m^6^A | Dot blot | Synaptic Systems | #202003 | Rabbit | 1:1000 |  |

**Supplementary Table 7. Sequence of primers used in the experiments**

|  | **Name** |  | **Oligo sequences (5’ to 3’)** |
| --- | --- | --- | --- |
| qPCR | LINC00989  ( NR_038826.1) | forward | ACACCGCCAGAAATTCCCTT |
|  |  | reverse | GCCCGTAGGCTAGCTGATTC |
|  | SENCR  (NR_038908.1) | forward | CCGTCTCTCCGCATTCTCC |
|  |  | reverse | TTACCTTGTCCACGCTCTCC |
|  | PROX1-AS1  (NR_037850.2) | forward | CAAAGGGGCTCACAGGACTT |
|  |  | reverse | GAAAGTGCCCCTGTTGGTCT |
|  | LINC00892  ( NR_038461.1) | forward | TCCGAGAGGCAGCTTGATTC |
|  |  | reverse | TAGCGGCAGAGCTGAGAGTA |
|  | LINC00504 F1  ( NR_126435.1) | forward | GGGAACTCTGGCCAAGCTAA |
|  |  | reverse | GCAGCCTCCAGGGTTAATCA |
|  | KIF25-AS1  ( NR_103750.1) | forward | CGTCATCAGCCTCAAATGCC |
|  |  | reverse | CACTCATCGCCACATCACCT |
|  | LINC01270  ( NR_034124.1) | forward | CTTGTCTGCTTTTGGAGCCG |
|  |  | reverse | TGGAGAGTCTGAGACAGCGT |
|  | MAP3K14  ( NR_024435.2 ) | forward | ACACCGCAGGTATCAGTAGC |
|  |  | reverse | TGGGAGCTCTTGAGTTGGTG |
|  | LINC00659  ( NR_046224.1) | forward | CTTGGGATCAGCCGAGCTTT |
|  |  | reverse | TTCCTTCGAGTCACTGAGATGC |
|  | VPS9D1-AS1  ( NR_036480.1) | forward | GTCGGCTCTACCACTGTTACT |
|  |  | reverse | AGCACGTCCTTGTCTCTGTA |
|  | LINC00200  ( NR_015376.2) | forward | TGTGCTCCGTACATCACACC |
|  |  | reverse | GCGGAAGGTTTTCCATGCAG |
|  | UCA1  ( NR_015379.3) | forward | ATGCACCCTAGACCCGAAAC |
|  |  | reverse | CTCCGGACTGCTTCAAGTGT |
|  | WASIR2  ( NR_130735.1) | forward | TCCGCGATGAGGACATCTCT |
|  |  | reverse | TTCCTCCTCCTTTCCCCGTA |
|  | 18S  ( M10098.1) | forward | ACAGGATTGACAGATTGA |
|  |  | reverse | TATCGGAATTAACCAGACA |
|  | FTO  (NM_001363894.1 ) | forward | TTCACCAGCATAGTATAGTT |
|  |  | reverse | AGTCTCCAATGTCATCAG |
|  | GAPDH  (NM_002046.4) | forward | CTCTGCTCCTCCTGTTCGAC |
|  |  | reverse | GCCCAATACGACCAAATCC |
|  | ABL  (NM_X16416) | forward | GGTACTCCATGGCTGACGAGAT |
|  |  | reverse | GAGTTCATGACCTACGGGAACCT |
|  | CyclinD1  ( NM_053056.3) | forward | ATCAAGTGTGACCCGGACTG |
|  |  | reverse | CTTGGGGTCCATGTTCTGCT |
|  | ITGA2  ( NM_002203.4) | forward | CAACATGAGCCTCGGCTTGA |
|  |  | reverse | CACAGAGGACCACATGTGAGAA |
|  | COL6A1  ( NM_001848.3) | forward | CATCAGCCAGACCATCGACA |
|  |  | reverse | ATTCGAAGGAGCAGCACACT |
|  | PDGFRA  ( NM_006206.6) | forward | TGCGGAATAACATCGGAGGA |
|  |  | reverse | GATTAGGCTCAGCCCTGTGA |
|  | PKN1  ( NM_213560.3) | forward | GAGCAGGAGCTGGAGAGTG |
|  |  | reverse | GCTGCTGCTGTACCCCG |
|  | F2R  ( NM_001992.5) | forward | CCCGCAGGCCAGAATCAAAA |
|  |  | reverse | TCATTGGGGTTCCTGAGAAGAA |
|  | HSP90AB1  ( NM_001271969.1) | forward | CCACTTGGCAGTCAAGCACT |
|  |  | reverse | GGAGCCCGACGAGGAATAAA |
| Cloning | SENCR16 |  | GTTTAAGCAGTGTGGAGATAT |
|  | SENCR785 |  | CAGAGGAGATCCAAGTCAATT |
|  | SENCR1238 |  | CCCACTGGAATTATTCAATAT |
|  | PROX1-AS1-7 |  | GAGGCTGGGAGCAGAAATAAT |
|  | PROX1-AS1-3299 |  | CAGCAGATTTACGGCAAATAT |
|  | PROX1-AS1-1277 |  | CAGCAGCTGTCGTGCTAATAA |
|  | LN892-370 |  | CTCAGCACCTGGCTCACATTA |
|  | LN892-619 |  | GGGTGTATAACACACATAATT |
|  | LN892-576 |  | CTTTGCATTGAACACTTTAAA |
