# Supplementary material for: The RNA N6-methyladenosine methylome coordinates long non-coding RNAs to mediate cancer drug resistance by activating PI3K signaling
Source: Cell Death Dis. 2025 Nov 7;16(1):804. doi: 10.1038/s41419-025-08045-6 (PMC12594950; doi:10.1038/s41419-025-08045-6)
Supplement: Supplementary file 2 — Uncropped gel images [file 41419_2025_8045_MOESM2_ESM.pdf]

Figure 1F

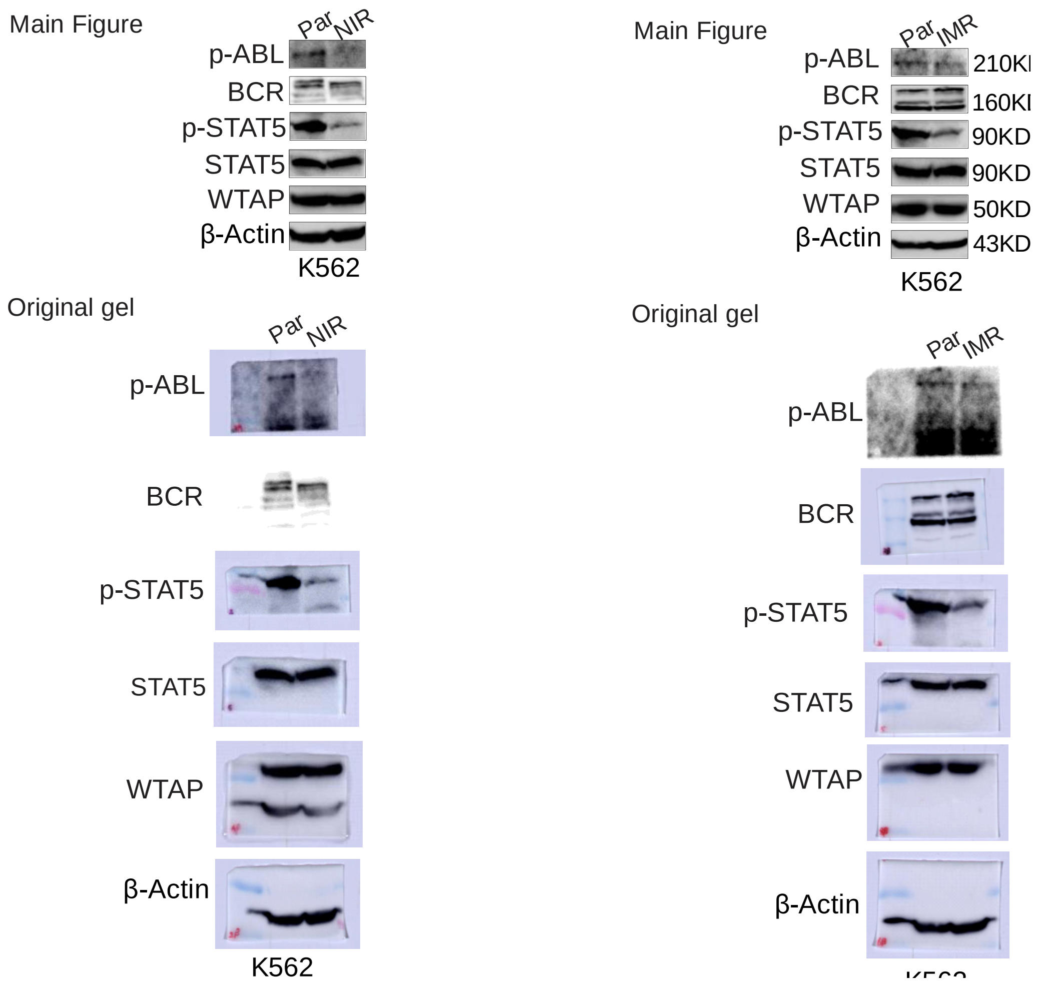
Main Figure
Par
NIR
p-ABL
BCR
p-STAT5
STAT5
WTAP
β-Actin
K562
Main Figure
Par
IMR
p-ABL
BCR
p-STAT5
STAT5
WTAP
β-Actin
210K
160K
90KD
90KD
50KD
43KD
K562
Original gel
Par
NIR
p-ABL
BCR
p-STAT5
STAT5
WTAP
β-Actin
K562
Original gel
Par
IMR
p-ABL
BCR
p-STAT5
STAT5
WTAP
β-Actin

Figure 1F

Main Figure

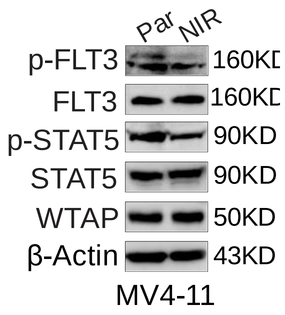

Original gel

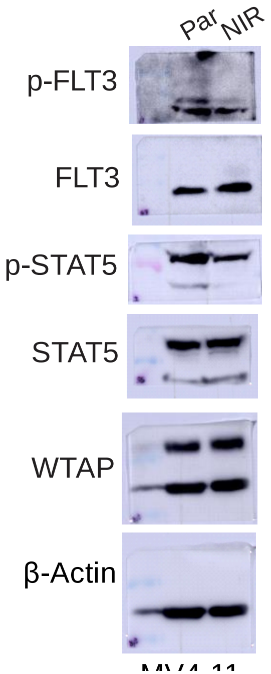

Figure 1F

Main Figure

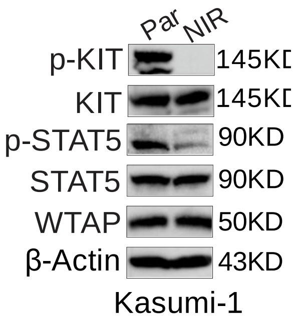

Original gel

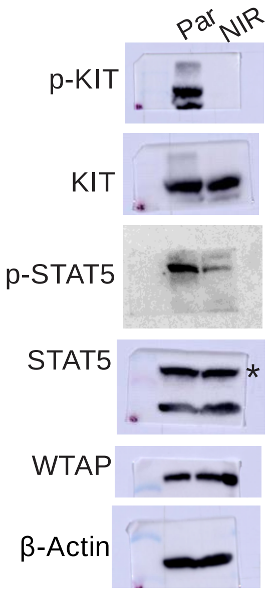

Figure 4A

Main Figure

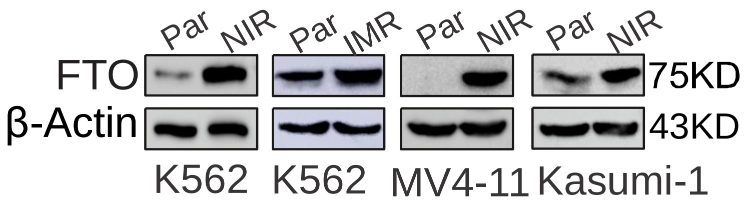

Submitted figure

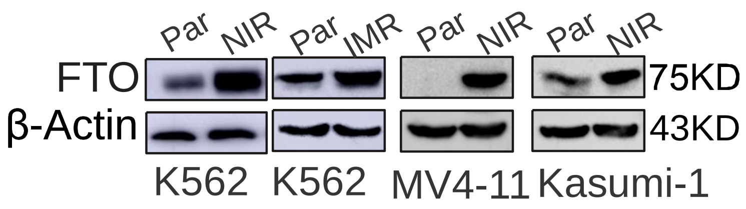

Revised figure

Original gel

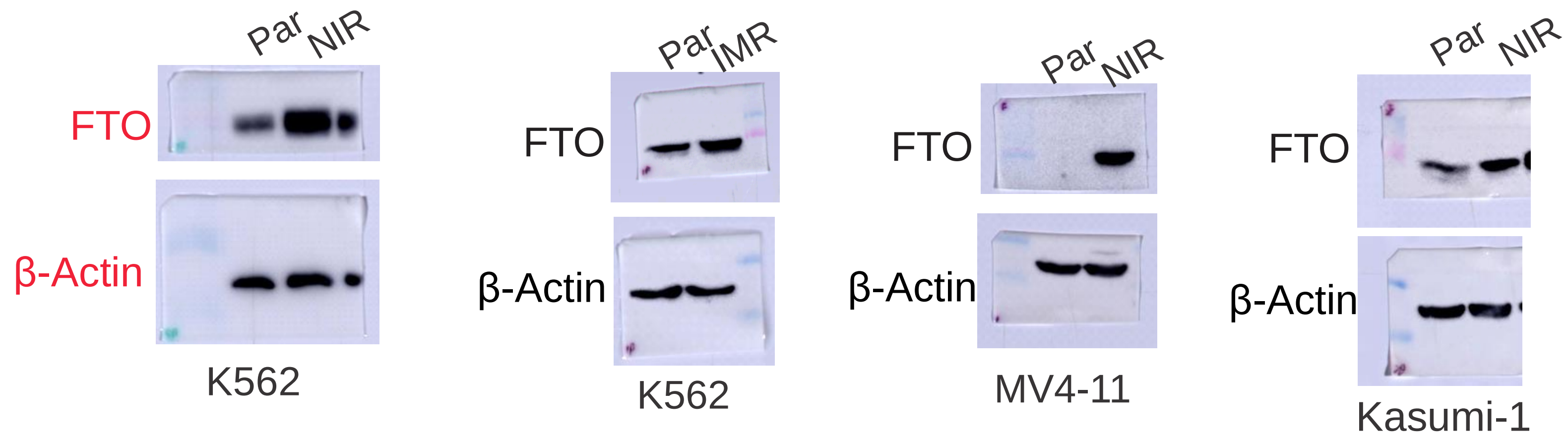

Figure 4E

Main Figure

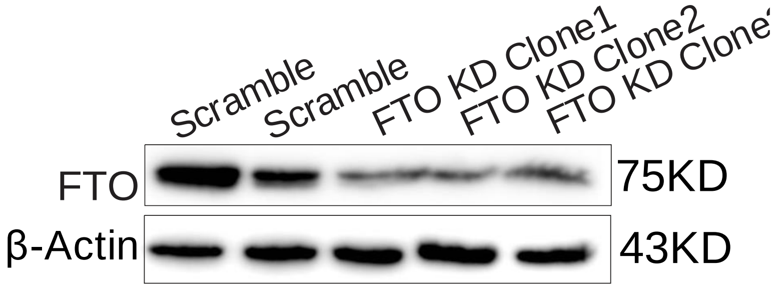

Original gel

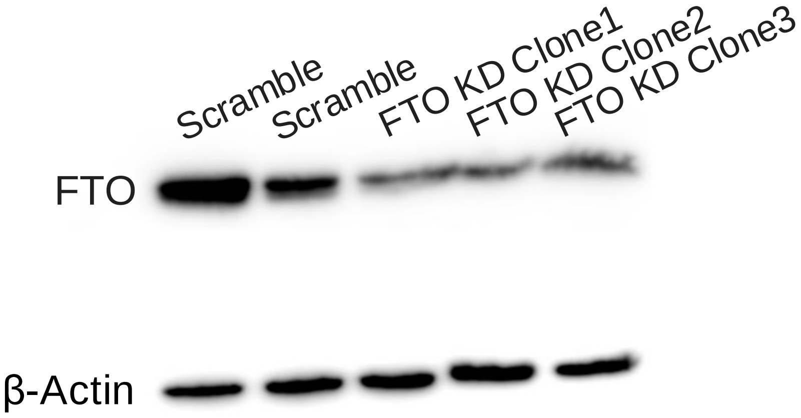

Fig. S7C

Main Figure

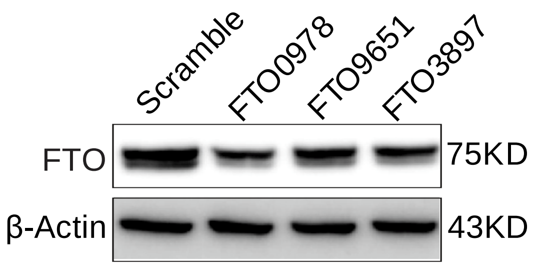

Submitted figure

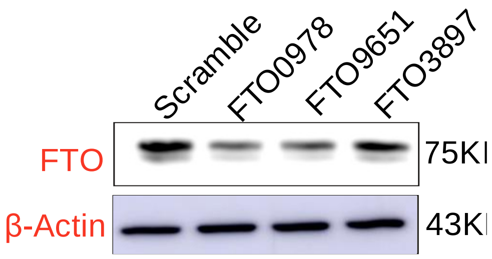

Revised figure

Original gel

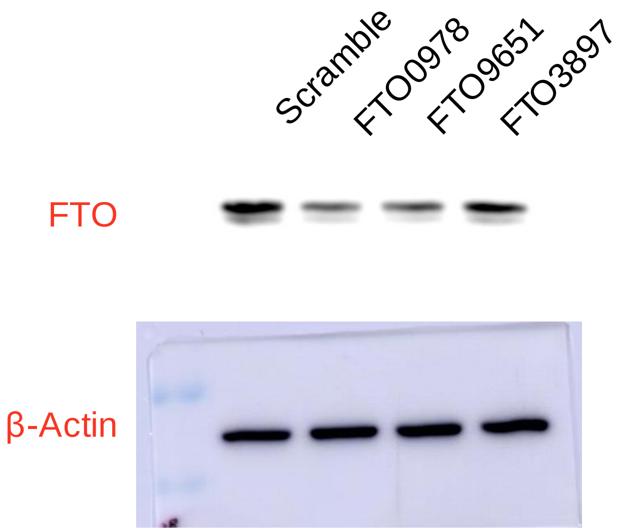

Fig. S7f

Main figure

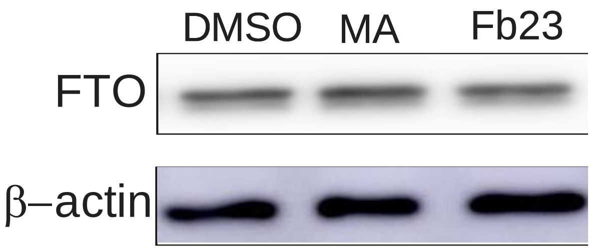

Original gel

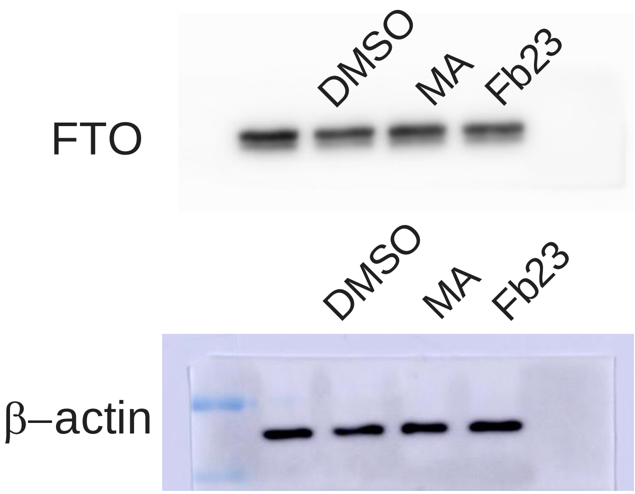

Figure 7E

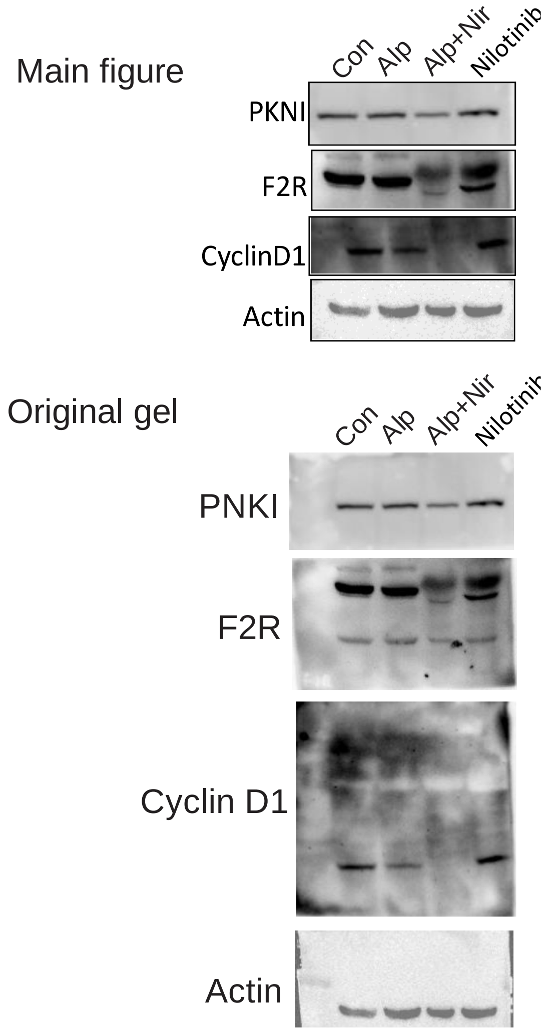
Main figure
Con
Alp
Alp+Nir
Nilotinib
PKNI
F2R
CyclinD1
Actin
Original gel
Con
Alp
Alp+Nir
PNKI
F2R
Cyclin D1
Actin
